# Last dental visit and severity of tooth loss: a machine learning approach

**DOI:** 10.1186/s13104-023-06632-4

**Published:** 2023-11-24

**Authors:** Rafael Aiello Bomfim

**Affiliations:** School of Dentistry, Federal University of Mato Grosso do Sul, Campo Grande, Brazil

**Keywords:** Tooth loss, Functional dentition, Severe tooth loss, Ageing, Machine learning, Structural equation modeling

## Abstract

The aims of the present study were to investigate last dental visit as a mediator in the relationship between socioeconomic status and lack of functional dentition/severe tooth loss and use a machine learning approach to predict those adults and elderly at higher risk of tooth loss. We analyzed data from a representative sample of 88,531 Brazilian individuals aged 18 and over. Tooth loss was the outcome by; (1) functional dentition and (2) severe tooth loss. Structural Equation models were used to find the time of last dental visit associated with the outcomes. Moreover, machine learning was used to train and test predictions to target individuals at higher risk for tooth loss. For 65,803 adults, more than two years of last dental visit was associated with lack of functional dentition. Age was the main contributor in the machine learning approach, with an AUC of 90%, accuracy of 90%, specificity of 97% and sensitivity of 38%. For elders, the last dental visit was associated with higher severe loss. Conclusions. More than two years of last dental visit appears to be associated with a severe loss and lack of functional dentition. The machine learning approach had a good performance to predict those individuals.

**Supplementary Information:**

The online version contains supplementary material available at 10.1186/s13104-023-06632-4.

## Introduction

The proportion of the Brazilian adults and elders population has increased due to an ongoing epidemiological transition process in Brazil and abroad [1, 2]. The Brazilian Unified Health System (SUS) has been providing universal coverage for the population since 2004 by the Family health strategy (FHS) [3, 4]. Despite little improvements in oral health and the declining prevalence of edentulism in the population, after the Brazilian Smiling Program, elders had not changed the DMF-T index. In 2003, it was 27,8 (92% of tooth loss), maintaining stable at 27,5 (91,9% of tooth loss) in 2010. Indeed, prevalence of edentulous changed from 53,2% in 2003 to 47,9% in 2010, corroborating that edentulism in the Brazilian population is a structural issue [5].

Despite advancements in Brazilian public policy, it is challenging to set priorities on the dental agenda for the whole population, especially concerning adults and elders, due to working time hours or mobility barriers. Primary Health Care (PHC) units need to equilibrate those who have already concluded dental treatment and need to be monitored through time on recalls intervals to prevent oral diseases [6–9]. Furthermore, it is necessary to take into account the social determinants of oral health [10, 11] that modulate access to oral health services in terms of structural, psychological and literacy barriers to a dental appointment [12, 13]. Educational-related inequalities in access to oral health services were found in several countries in elders [14]. Even in high-income countries, adults have neglected oral health, with 21% not attending any year in the past six years in Scotland [15]. A machine learning approach would help predict those adults and elders at higher risk for tooth loss and helps schedule a dental visit and establish masticatory function and quality of life. This approach has been tested with US data only for adults [16] and elders in Japan [17]. It would be great to organise dental agenda and set priorities. Tooth loss is a paramount public health concern for global health [18, 19] that impacts peoples’ quality of life and is influenced by socioeconomic position [20, 21].

The aims of the present study were to investigate last dental visit as a mediator in the relationship between socioeconomic status and lack of functional dentition/severe tooth loss and use a machine learning approach to predict those adults and elders at higher risk of tooth loss in Brazil.

## Methods

The data analysed in this study came from the 2019 National Health Survey (Pesquisa Nacional de Saúde - PNS), designed to have a nationally representative sample of the Brazilian population. The 2019 PNS is a cross-sectional household survey with a sampling process carried out in three stages. More detailed information can be found elsewhere [22]. The PNS data is freely available on the Brazilian Institute of Geography and Statistics (IBGE) website: https://www.ibge.gov.br/estatisticas/downloads-estatisticas.html.

### Tooth loss assessment

Tooth loss was measured by asking: “Have you lost any of your permanent upper teeth?“ Response options were (1) No; (2) Yes, I have lost (number) teeth; (3) Yes, I have lost all my upper teeth. The same question was asked for the lower permanent teeth. Upper and lower self-reported tooth loss count was analysed classified into two levels of severity, regardless of the tooth position: (1) Functional dentition, defined as a loss of up to 12 permanent teeth; and (2) Severe tooth loss considered the loss of 23-32 [18].

### Covariates

Gender (men or women); age; schooling was classified into formative years (0 to 8) and formal education (8 or more years) for both adults and elders. Income was dichotomized according to the official Brazilian minimum wage (MW) in 2019, into equal or higher than one per capita MW and less than one. Socioeconomic status was a latent variable measured by 2 indicators: schooling and income. The last dental visit was categorized into two groups: up to two years and more than two years. Officially named in Brazil as colour/race, the self-described race was assessed using five options according to the categories proposed by the Brazilian Institute of Geography and Statistics (IBGE): (1) white; (2) black; (3) yellow; (4) brown, and (5) indigenous. Self-reported smoking status was measured as those who never smoked and individuals who smoked in the past and/or are current smokers and use of dental floss (yes/no) only for adults due to the percentage of edentulous in the elders (almost half of the sample that does not have chance to use dental floss due to the loss of all teeth). Dental floss use was considered as an oral health behaviour and was stratified as (yes/no).

### Statistical analysis

**Fig. 1:**
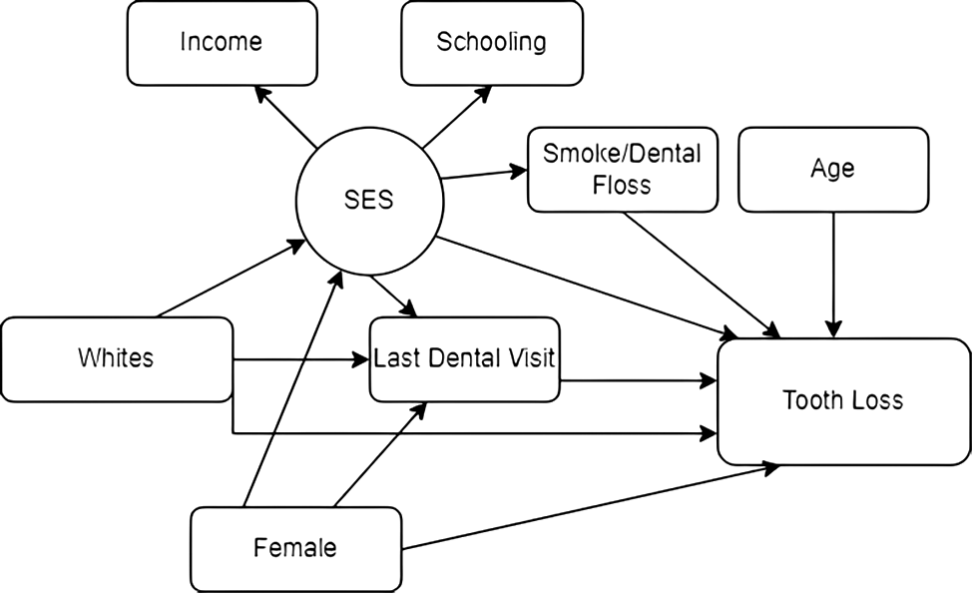
The social determinants of oral health adapted to individual-level predictors

Age-standardised estimates for functional dentition and severe tooth loss (including edentulous) were reported for each covariate. Data analysis for the Structural Equation Modelling (SEM) was performed using the Stata software version 14.2 (StataCorp LP, College Station, United States) using the survey module that considers the effects of stratification and conglomeration in estimating indicators and their precision measures. This method allows the simultaneous testing of complex interrelationships between variables in public health. The variables were tested using the social determinants of oral health [10] and Sisson’s model as theoretical explanations [11]. Figure 1 exemplifies the finals SEM models for adults and elders. Standardised coefficients (SCs) were interpreted as being a small association (SC < 0.10), medium association (SCs between 0.30 and 0.50), and a strong association (SC > 0.50) [23].

### Machine learning approach

The Extreme gradient boost algorithm (Xgboost), based on sequential models of decision trees, was used to predict the outcome for adults and elders. Previous research has shown this algorithm has the highest area under the receiver operating characteristic curve (AUC) than others [16]. Appendix 1 shows the strategy for tuning all hyperparameters and the grids created. All of the results presented here are from the test set. Finally, to assess the predictive performance of the trained algorithm, the AUC, accuracy, sensitivity and specificity were calculated.

Furthermore, we computed the importance of each covariate in predicting our study outcomes, one for the adults and the elders. For the first sensitivity analysis, we stratify our dataset into the five main regions in Brazil (a proxy for human development levels). Southeast and South regions have better development levels than Northeast and North and different concentrations of dental surgeons workforce in both public and private sectors [24]. We used R (R Foundation for Statistical Computing, Vienna, Austria) software for our machine learning approach. We followed the STROBE guidelines for human observational studies and the checklist for the artificial intelligence approach [25].

### Ethical aspects

The Brazilian Committee (Health National Council Committee) approved the Ethics in Human Research (protocol number 3.529.376).

## Results

Out of the 90,846 individual who have answered the questionnaire, the analytical sample comprised 65,803 adults, and 22,728 elders. Table 1 shows the sample characteristics. The age-standardised estimates of having a functional dentition were higher among white participants, men, earning above one minimum wage, higher schooling, non-smokers, for both adults and elders.

**Table 1:** Descriptive characteristics and proportions of Brazilians adults and elders and age-standardised prevalence of levels of tooth loss in Brazilian National Health Survey 2019 (*n* = 88,531)

Individual variables	*n* = 65,803	%	Presence of Functional dentition**		*n* = 22,728	%	Severe tooth loss **
			% (95% CI)				% (95% CI)
Individual variables	Adults (Prevalence = 89,5%)				Elders (Prevalence = 65,5%)		
**Racial Groups**							
Whites	22,508	41.3	91.4 (90.8; 92.0)		9901	50.5	60.1 (57.3; 62.9)
NonWhites*	43,295	58.7	88.0 (87.5; 88.5)		12,827	49.5	70.3 (68.2; 72.2)
**Sex**							
Female	34,334	52.2	87.8 (87.2; 88.4)		12,535	56.7	69.4 (67.3; 71.4)
Male	31,469	47.8	91.4 (90.9; 91.9)		10,193	43.3	59.4 (57.1; 61.5)
**Per capita Income**							
< 1 minimum wage	38,046	53.8	85.4 (84.8; 85.9)		10,250	41.7	75.2 (73.5; 76.8)
≥ 1 minimum wage	27,738	46.2	93.3 (92.8; 93.7)		12,475	58.2	57.9 (55.3;60.3)
**Schooling**							
< 8 years	25,689	34.9	83.8 (83.2; 84.4)		16,414	70.1	71.2 (69.1; 73.2)
≥ 8 years	40,114	65.1	95.0 (94.7; 95.2)		6314	29.9	38.9 (36.0; 41.9)
**Smoke**							
yes/in the past	21,162	32.3	86.5 (85.9; 87.1)		11,117	50.3	65.8 (63.4; 67.3)
never	44,641	67.7	91.5 (91.0; 92.0)		11,611	49.7	65.4 (63.2; 68.3)
**Dentist visit last 24 months**							
yes	45,081	71.1	91.9 (91.5; 92.4)		9942	47.4	44.8 (42.0; 47.6)
no	20,722	28.9	85.0 (84.3; 85.6)		12,786	52.6	77.2 (75.5; 78.7)
**Age Groups**							
**18–34**	24,115	40.8	99.4 (99.2; 99.5)	**60–69**	24,247	56.3	38.4 (36.9; 39.9)
**35–44**	18,033	25.8	95.3 (94.8; 95.8)	**70–79**	13,209	30.1	59.0 (57.0; 61.0)
**45–59**	23,655	33.4	74.4 (73.4; 75.4)	**80 +**	6098	13.6	78.5 (74.9; 81.7)

* Browns/Blacks/Indigenous/Asian

** Age-standardized

Table 2 showed the direct, indirect and total effects of SES, last dental visit, race and gender on the tooth loss outcomes, adjusted for smoke and use of dental floss (only for the adult’s population). Socioeconomic statuses SC = 0.20 (CI 95% 0.16; 0.23) was directly associated to the presence of functional dentition in adults and with last dental visit up to 2 years SC = 0.62 (CI 95% 0.56; 0.68). The last dental visit mediated the effect of race on tooth loss, favouring the Whites. Concerning the elders, better SES SC = − 0.71 (CI 95% -0.79; -0.62) and last dental visit up to 2 years SC = − 0.17 (CI 95% -0.20; -0.14) were associated with less severe tooth loss. The Last dental visit up to 2 years mediated the effect of race SC = − 0.15 (CI 95% -0.16; -0.14) and SES SC = − 0.17 (CI 95% -0.20; -0.14) on severe tooth loss, favouring the Whites and the higher socioeconomic position. The fit indices of the models were considered appropriate and are presented in Table 2.

**Table 2:** Structural Equation models with association of SES, race, gender and LDV on tooth loss, Brazilian National health survey, 2019

	Adults (Functional dentition)*				Elders (Severe Loss)*		
**Direct Effects**	SC	95% CI	*p*		SC	95% CI	*p*
**Last Dental visit < 2 years**							
LDV → tooth loss	0.01	(-0.01/0.02)	0.09		─0.17	(-0.20/-0.14)	0.001
**Sócio-demografic**							
Female → tooth loss	─0.04	(-0.05/-0.03)	< 0.001		0.10	(0.08/0.13)	< 0.000
SES → Tooth loss	0.20	(0.16/0.23)	< 0.001		─0.71	(-0.79/-0.62)	< 0.001
Whites → Tooth loss	─0.03	(-0.04/-0.02)	< 0.001		0.07	(0.05/0.10)	< 0.001
**Last Dental visit (LDV) < 2 years**							
SES → LDV	0.62	(0.56/0.68)	< 0.001		1.00	(0.92/1.07)	< 0.001
Whites → LDV	─0.03	(-0.04/-0.01)	< 0.001		─0.02	(-0.04/0.01)	0.11
Female → LDV	0.10	(0.08/0.11)	< 0.001		0.02	(-0.01/0.04)	0.08
**Indirect Effects**							
**Via Last dental visit < 2 years**							
SES→ tooth loss	0.01	(-0.01/0.01)	0.10		─0.17	(-0.20/-0.14)	< 0.001
Whites→ tooth loss	0.05	(0.04/0.06)	< 0.001		─0.15	(-0.20/-0.14)	< 0.001
Female→ tooth loss	─0.00	insignificant			0.02	(0.01/0.03)	0.008
**Via SES**							
Whites → LDV	0.13	(0.12/0.15)	< 0.001		0.18	(0.15/0.20)	< 0.001
Female → LDV	─0.01	(-0.01/0.01)	0.3		─0.01	(-0.02/0.01)	0.7
**Total Effects**							
**Sócio demographic**							
LDV → tooth loss	0.01	(-0.01/0.02)	0.08		─0.17	(-0.20/-0.14)	< 0.001
Whites → tooth loss	0.02	(0.01/0.03)	< 0.001		─0.08	(-0.10/-0.05)	< 0.001
Female → tooth loss	─0.04	(-0.05/-0.03)	< 0.001		0.12	(0.10/0.15)	< 0.001
SES → tooth loss	0.21	(0.17/0.23)	< 0.001		─0.88	(0.02/0.06)	< 0.001
Smoke → tooth loss	─0.04	(-0.05/-0.03)	< 0.001		0.04	(-0.95/-0.80)	< 0.001
Goodness of Fit							
RMSE	0.08				0.07		
CFI	0.81				0.92		
TLI	0.80				0.80		
Chi2	< 0.05				< 0.05		
SRMR	0.06				0.04		

* adjusted for age and smoke status (elders) and age, smoke and dental floss (adults)

RMSE- root mean square error, CFI - Comparative fit index; TLI- Tucker lewis index, SRMR Standardized root mean squared residual

Table 3 shows the machine learning approach. For the adults, our trained Xgboost algorithm showed that the accuracy was 90%, predicting those with a lack of functional dentition, which means that in every ten adults, we correct classified nine in the Brazilian context. The AUC was 90%, sensitivity 38% and specificity 97%, and age was the main contributor. For the elders, our trained algorithm showed an accuracy of 70%. It means that we corrected classified seven in ten elders. The AUC was 76%, sensitivity 68% and specificity of 73%, and interestingly, the last dental visit was the main contributor. Sensitivity analysis by regions (a proxy for the human development levels) showed increased AUC by 91% and accuracy of 93% in the Southeast region in adults. Elders showed the same patterns. Indeed, regions changed the importance of individual variables in the machine learning approach (Appendix 1). In the second sensitivity analysis, with under sample the majority class in the training set, no differences were found in the testing set in terms of collected metrics.

**Table 3:** Machine learning approach and metrics for adults and elders in the whole country and divided by geographic regions

Collect metrics	Adults (Lack of Functional Dentition)			Elders (Severe Loss)
Sensitivity	0.38		Sensitivity	0.66
Specificity	0.97		Specificity	0.74
Acuraccy	0.90(CI95% ± 0.01)		Acuraccy	0.70(CI95% ± 0.01)
AUC	0.90 (CI95% ± 0.01)		AUC	0.77(CI95% ± 0.01)
**Three Main contributors**	importance*		**Three Main contributors**	importance*
Age	0.58		Last dental Visit**	0.43
Schooling	0.18		Schooling	0.22
Dental Floss**	0.13		Age	0.20
**Metrics by region**				
**Southwest**	***n*** ** = 13,610**			***n*** ** = 5825**
Sensitivity	0.39		Sensitivity	0.78
Specificity	0.98		Specificity	0.66
Acuraccy	0.93(CI95% ± 0.01)		Acuraccy	0.73(CI95% ± 0.03)
AUC	0.91(CI95% ± 0.01)		AUC	0.80(CI95% ± 0.02)
**Main contributor**			**Main contributor**	
Age	0.49		Dental Visit	0.38
**South**	***n*** ** = 7969**			***n*** ** = 3307**
Sensitivity	0.39		Sensitivity	0.80
Specificity	0.97		Specificity	0.60
Acuraccy	0.91(CI95% ± 0.01)		Acuraccy	0.71(CI95% ± 0.03)
AUC	0.91(CI95% ± 0.01)		AUC	0.77(CI95% ± 0.03)
**Main contributor**			**Main contributor**	
Age	0.49		Dental Visit	0.40
**Widwest**	***n*** ** = 7808**			***n*** ** = 2373**
Sensitivity	0.30		Sensitivity	0.72
Specificity	0.97		Specificity	0.66
Acuraccy	0.90(CI95% ± 0.02)		Acuraccy	0.69(CI95% ± 0.04)
AUC	0.90 (CI95% ± 0.02)		AUC	0.76(CI95% ± 0.04)
**Main contributor**			**Main contributor**	
Age	0.55		Dental Visit	0.41
**Northwest**	***n*** ** = 22,966**			***n*** ** = 7736**
Sensitivity	0.41		Sensitivity	0.59
Specificity	0.96		Specificity	0.80
Acuraccy	0.88 (CI95% ± 0.01)		Acuraccy	0.71(CI95% ± 0.02)
AUC	0.88 (CI95% ± 0.01)		AUC	0.75(CI95% ± 0.02)
**Main contributor**			**Main contributor**	
Age	0.62		Dental Visit	0.38
**North**	***n*** ** = 13.450**			***n*** ** = 3487**
Sensitivity	0.29		Sensitivity	0.58
Specificity	0.97		Specificity	0.75
Acuraccy	0.89 (CI95% ± 0.01)		Acuraccy	0.67(CI95% ± 0.03)
AUC	0.89 (CI95% ± 0.01)		AUC	0.71(CI95% ± 0.03)
**Main contributor**			**Main contributor**	
Age	0.69		Age	0.38

* importance range from 0 to 1. The main gain on information, the more important the predictor role on the outcome

** Possibly modified factors to be adressed for Primary health care workers

## Discussion

This present research showed two critical findings. First, the last dental visit was a mediator between SES and severe tooth loss in elders. Secondly, in the machine learning approach, the trained Xgboost algorithm had good metrics predicting the lack of functional dentition in adults, with age, schooling and dental floss as the main contributors, indicating that it should be used for FHS in Brazil.

It is of fundamental importance to identify adults without functional dentition to schedule an appointment in primary health care. This can help with the principle of equity, giving more attention to those who need it most, considering that the absence of functional dentition affects the quality of life of adults and elders [26–30]. The algorithm trained for the Brazilian context is easy to use and with only a few input variables, generally collected in one visit by community health workers(CHW), an accuracy of 90% was achieved, that is, getting 90 out of 100 adults right. Moreover, for elders, identifying those with a higher risk for severe tooth loss in the adscript area of FHS could improve the planning and management for Dental Specialties Centers in Brazil. They are responsible for secondary care (prosthesis, for example) and give agility and resolution for elders to reestablish masticatory function and quality of life. For example, even without a dental consultation by a dentist in the PHC, the Family Health Strategy could use the trained algorithm, and target all individuals at higher risk for tooth loss. If correctly implemented, the work process has potential to be changed in the country. And the consultations could be target on those who need it more to prevent tooth loss. The algorithm can function as a support for care coordination.

Some structural barriers and contextual factors could be related to tooth loss, as recent evidence on the commercial determinants of health and its association with oral health [31]. The last dental visit was a mediator of SES for severe loss in elders and a borderline in adults for functional dentition (indirect effect *p* = 0.10). However, it is unrealistic for the health sector to be able to change people’s socioeconomic condition. Identifying those with more than 2 years of last dental visit, carrying out an active search in FHS and scheduling appointments, maybe with help of machine learning, could help adults and elders to prevent tooth loss. Future research needs to explore the role of contextual characteristics at different levels. An important finding of the machine learning approach for adults and elders was that schooling is one of the three most crucial predictors of lack of functional dentition/severe tooth loss in the Brazilian context. In a multicountry study, there were significant education-related inequalities in using oral health services by elders [14], corroborating our findings. Monitoring these inequalities is critical to planning and delivering dental services, organizing dental agenda, and setting priorities.

### Limitations

This study has some strengths and limitations that should be acknowledged. The strengths were using a nationally representative sample of Brazilian adults and elders, the use of two definitions of tooth loss internationally to compare populations [18, 33–35] and promote machine learning as a potential path to set priorities on the dental agenda in the Unified health system in Brazil and care coordination.

One limitation was the self-reported nature of tooth loss assessment that may lead to information bias, but previous research has shown a good concordance between self-reported tooth loss and clinical evaluation in national surveys [32]. Another limitation of our study is its cross-sectional design, which may not establish a temporal relationship between exposures and outcomes.

Policymakers should use implementation science frameworks [36, 37] to train community health workers and measure organisational readiness [38, 39] for using the machine learning approaches. The algorithm could be retrained at local levels and improve current performance in each context of primary care in Brazil. Future works using tooth loss and machine learning approaches need to be further investigated.

In conclusion, more than two years of last dental visit appears to be associated with a severe loss in elders and lack of functional dentition in adults. The machine learning approach had a good performance to predict those individuals. The Family Health Strategy should use the trained algorithm to target those individuals in the Brazilian context and set priorities on dental agendas.

### Electronic supplementary material

Below is the link to the electronic supplementary material.

Appendix 1

## Data Availability

The data that support the findings of this study are openly available in [Brazilian Institute of Geography and Statistics (IBGE)] at [https://www.ibge.gov.br/estatisticas/downloads-estatisticas.html].
