# Supplementary material for: Last dental visit and severity of tooth loss: a machine learning approach
Source: BMC Res Notes. 2023 Nov 24;16:347. doi: 10.1186/s13104-023-06632-4 (PMC10668397; doi:10.1186/s13104-023-06632-4)
Supplement: Supplementary file 1 — Appendix 1 [file 13104_2023_6632_MOESM1_ESM.docx]

**Appendix 1 –**

**Strategy for tunning hiperparameters with all dataset.**

**Older Adults**

Graphs for older adults selecting the best AUC

Step 1 – Tuning trees and learning rate


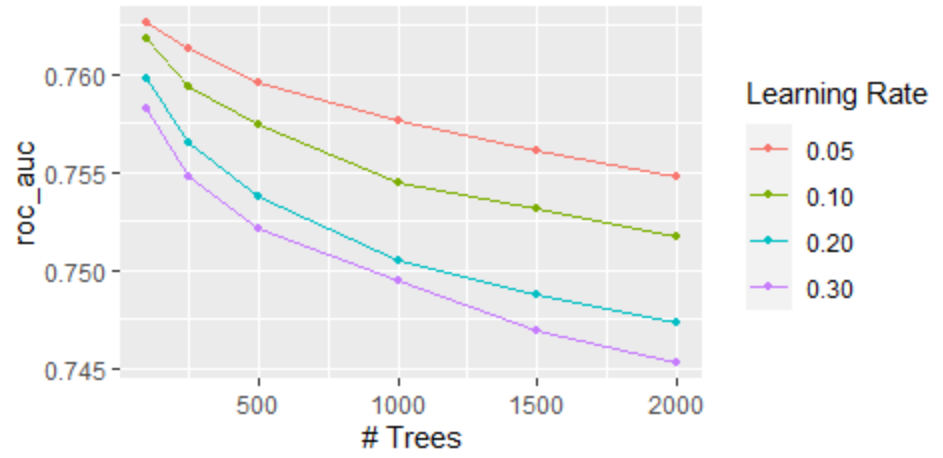


Step 2 - **Tunning tree Deph and Minimal node size**


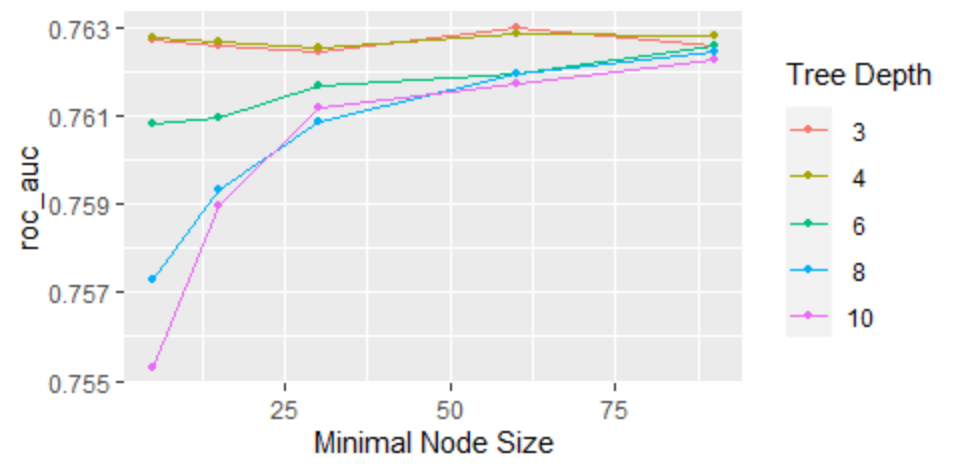


Step 3- **Tunning minimal loss reduction**


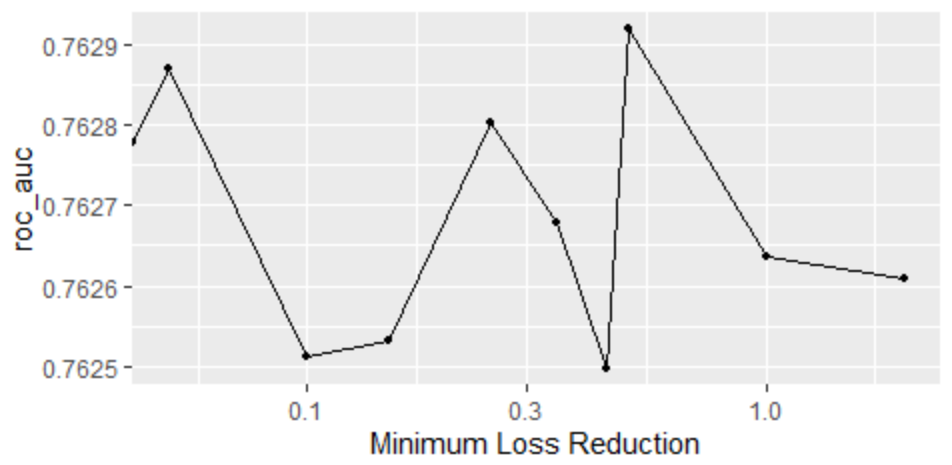


Step 4 – **Tunning mtry and Sample Size**


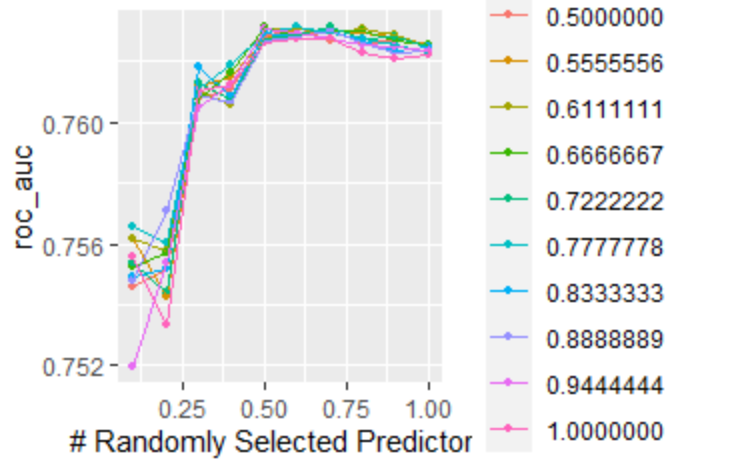


**Step 5 – Learning rate and trees final again**

**
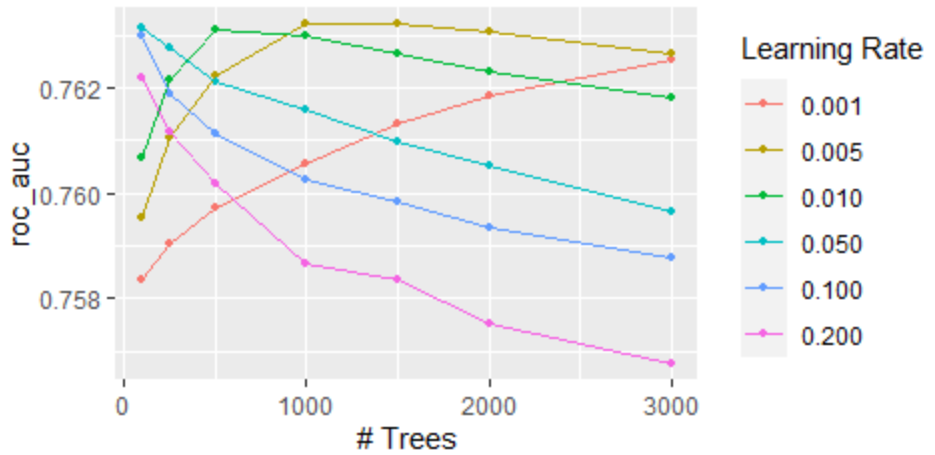
**

**Step 6 - Collect_metrics in the test set.**

.metric .estimator .estimate .config

*<chr>* *<chr>* *<dbl>* *<fct>*

1 accuracy binary 0.698 Preprocessor1_Model1

2 f_meas binary 0.686 Preprocessor1_Model1

3 ppv binary 0.712 Preprocessor1_Model1

4 npv binary 0.686 Preprocessor1_Model1

5 sens binary 0.661 Preprocessor1_Model1

6 spec binary 0.735 Preprocessor1_Model1

7 roc_auc binary 0.771 Preprocessor1_Model1

Step 7 - Importance of predictors


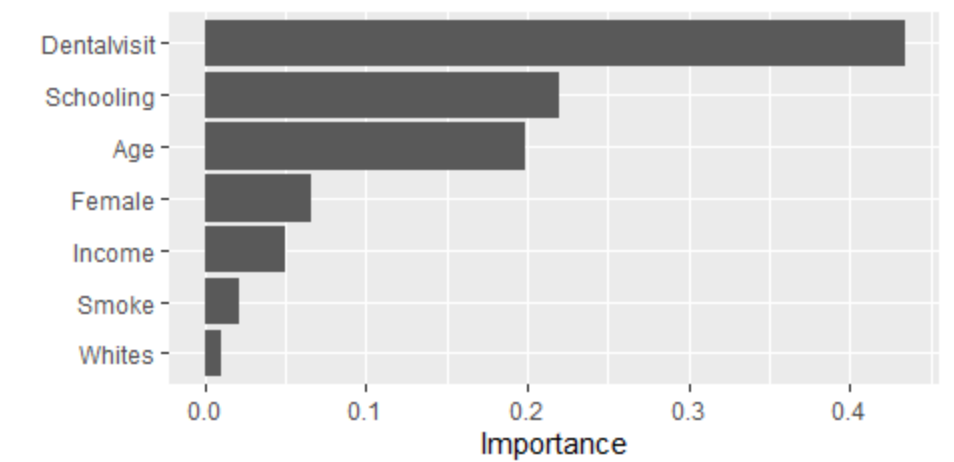


Step 8 – Roc –Curve


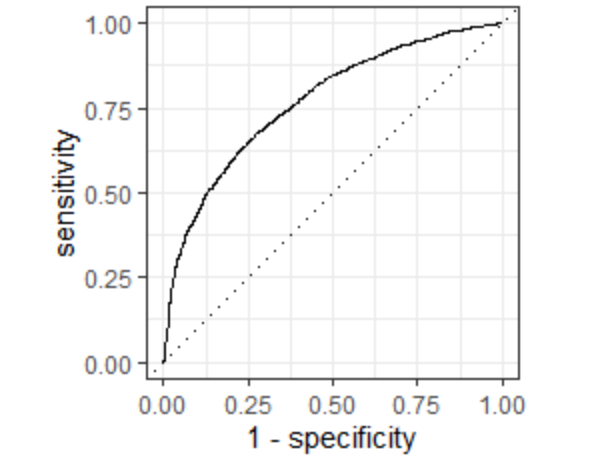


**Adults**

**Step 1 -** Tunning trees and learning rate

**
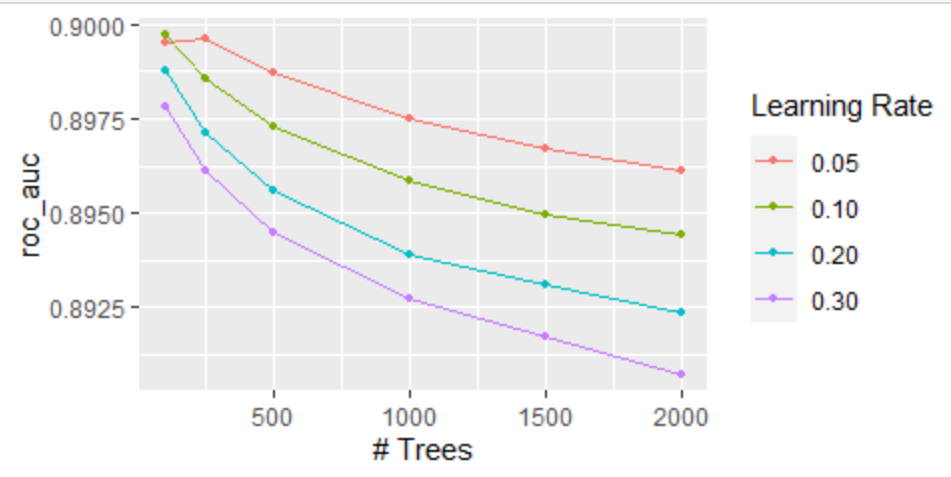
**

**Step 2 - Tunning tree Deph and Minimal node size**

**
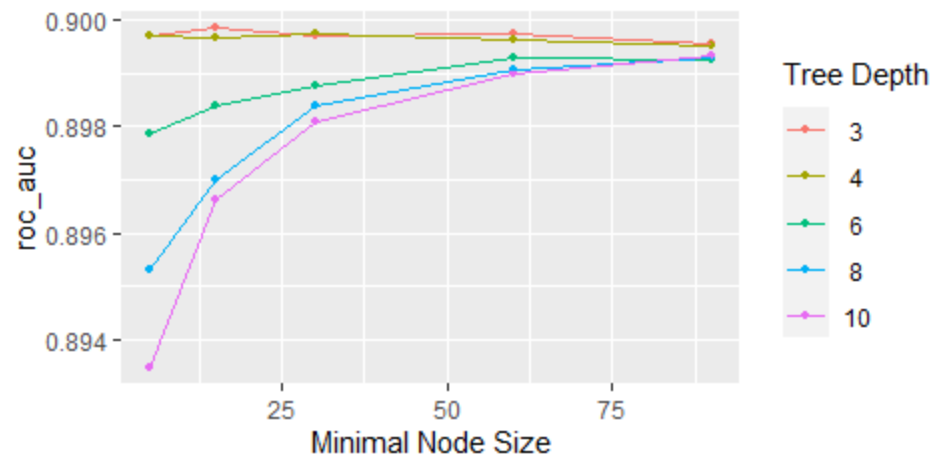
**

**Step 3 – tunning minimal loss reduction**

**
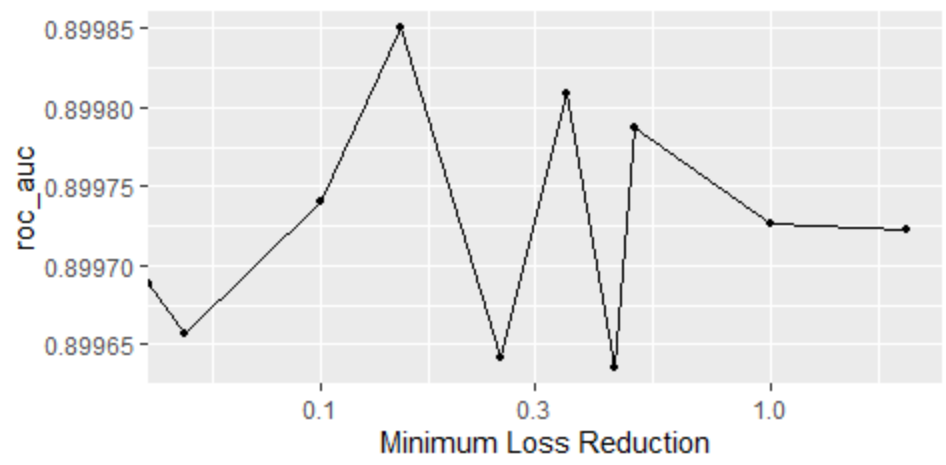
**

**Step4 – tunning Mtry and sample size**

**
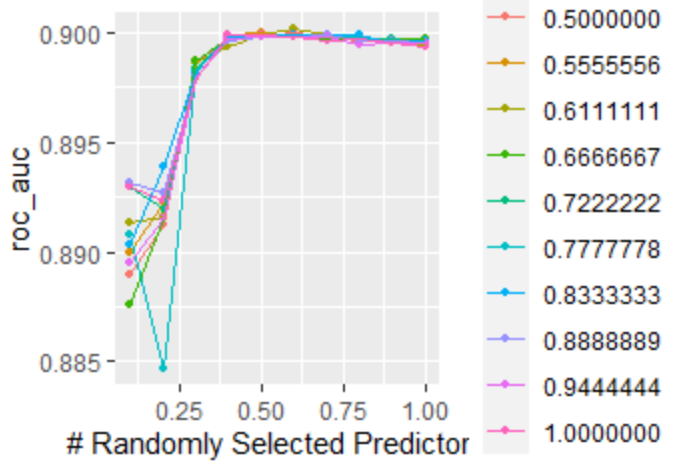
**

**Step 5 – Learning rate and trees final again**

**
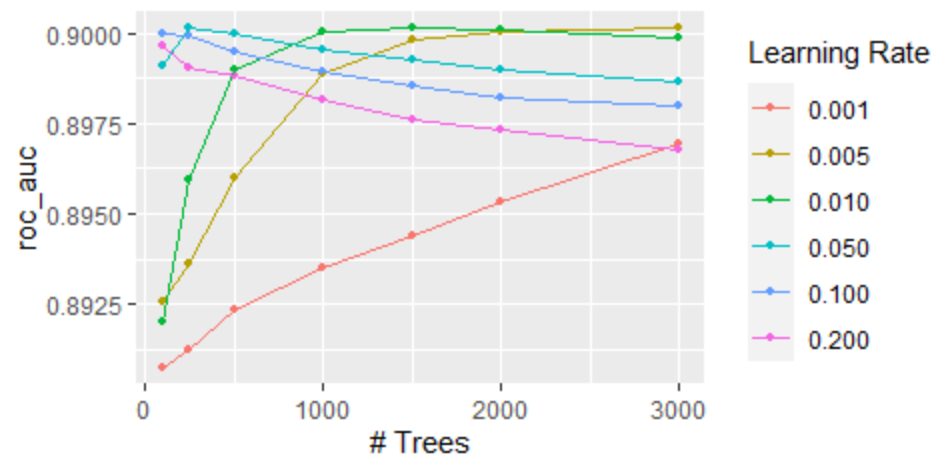
**

**Step 6 - Collect_metrics in the test set. ( to detect lack of FD)**

.metric .estimator .estimate .config

*<chr>* *<chr>* *<dbl>* *<fct>*

1 sens binary 0.379 Preprocessor1_Model1

2 spec binary 0.971 Preprocessor1_Model1

3 accuracy binary 0.898 Preprocessor1_Model1

4 ppv binary 0.632 Preprocessor1_Model1

5 f_meas binary 0.463 Preprocessor1_Model1

6 npv binary 0.918 Preprocessor1_Model1

7 roc_auc binary 0.897 Preprocessor1_Model1

**Step 7 - Importance of predictors**


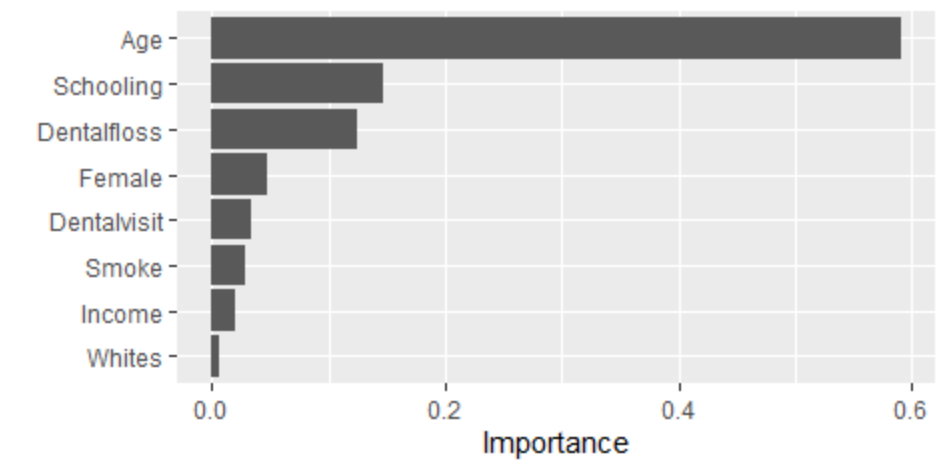


Step 8 - Roc Curve


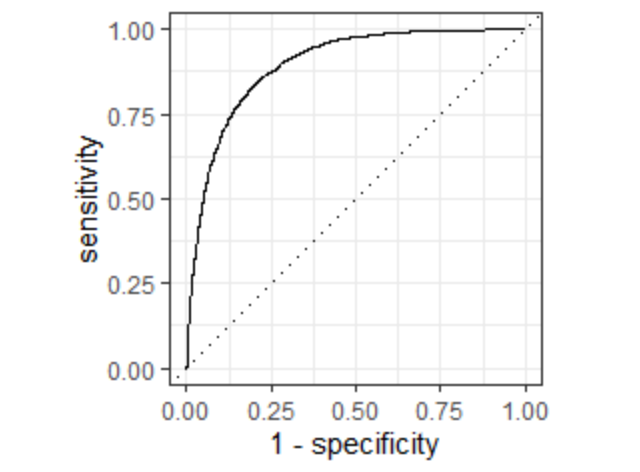


**Sensitivity Analysis 1**

**Adults**

**North Region.**

Step 1 - Tunning trees and learning rate


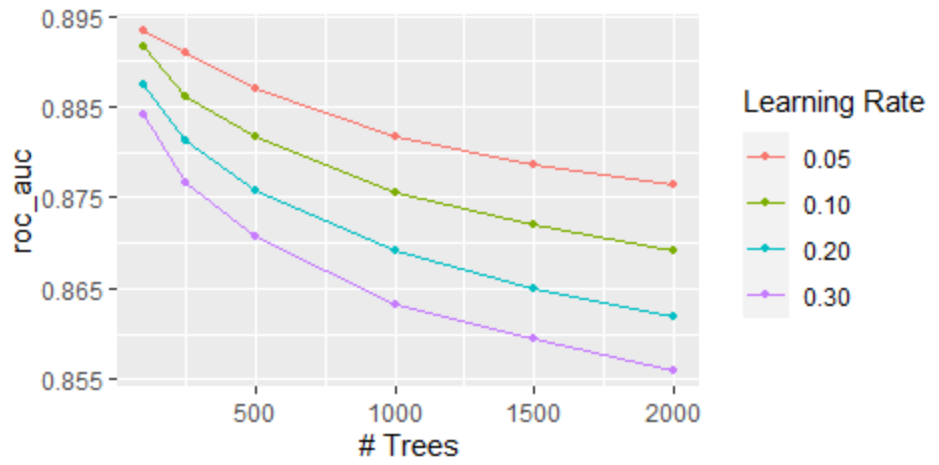


Step 2 Tunning tree Deph and Minimal node size


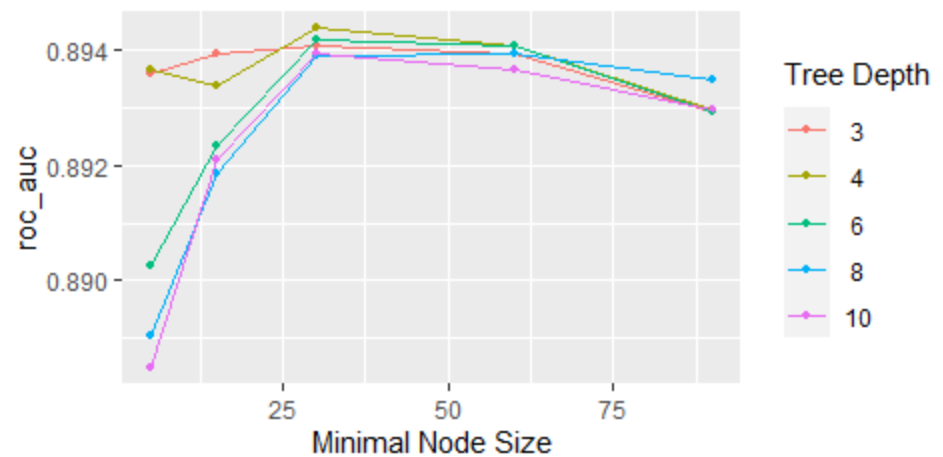


Step 3 tunning minimal loss reduction


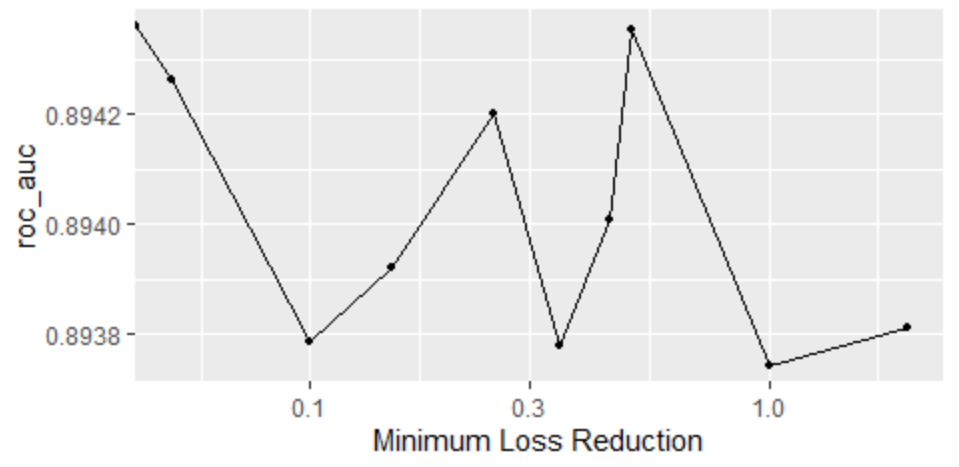


Step 4 Tunning mtry and Sample Size


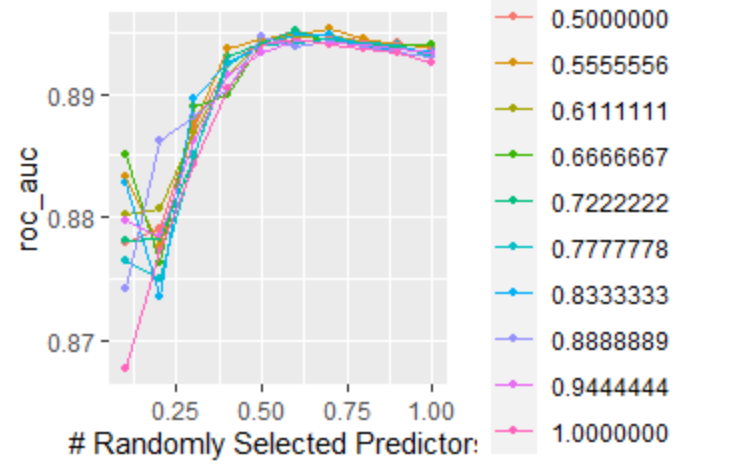


Step 5- Learning rate and trees final again


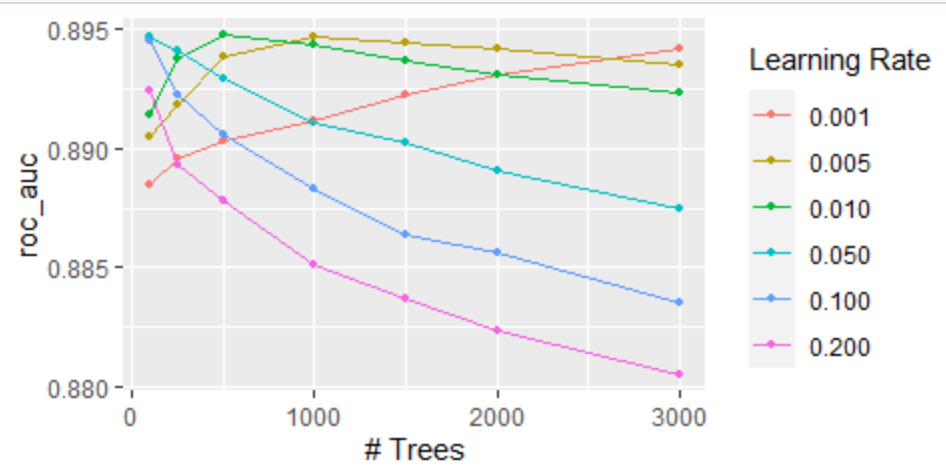


**Step 6 - Collect_metrics in the test set.**

1 sens binary 0.286 Preprocessor1_Model1

2 spec binary 0.968 Preprocessor1_Model1

3 accuracy binary 0.888 Preprocessor1_Model1

4 ppv binary 0.548 Preprocessor1_Model1

5 f_meas binary 0.376 Preprocessor1_Model1

6 npv binary 0.910 Preprocessor1_Model1

7 roc_auc binary 0.887 Preprocessor1_Model1

>

**Step 7 - Importance of predictors**


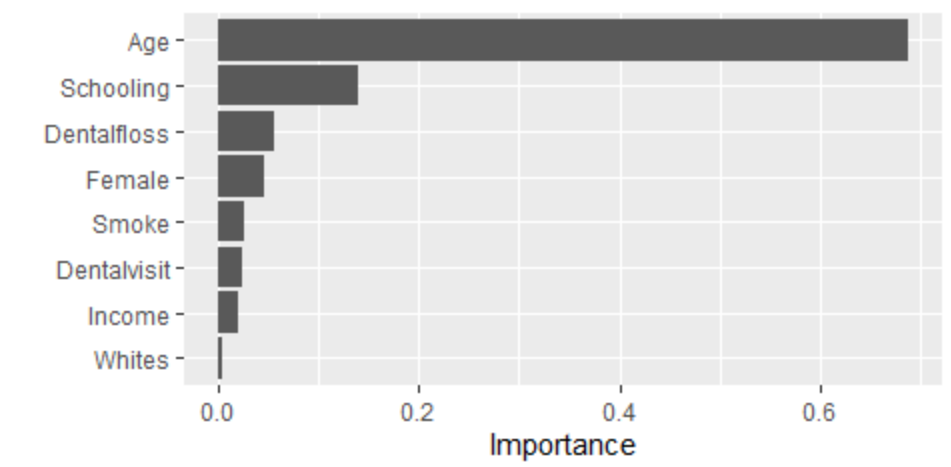


**Step 8 – Roc –Curve**


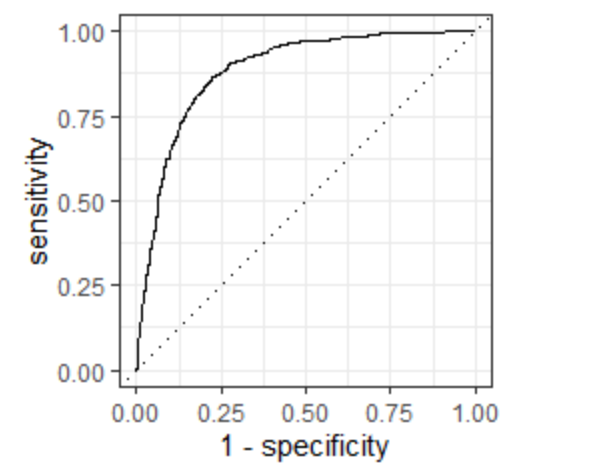


**Southeast Region**

Step 1 - Tunning trees and learning rate

**
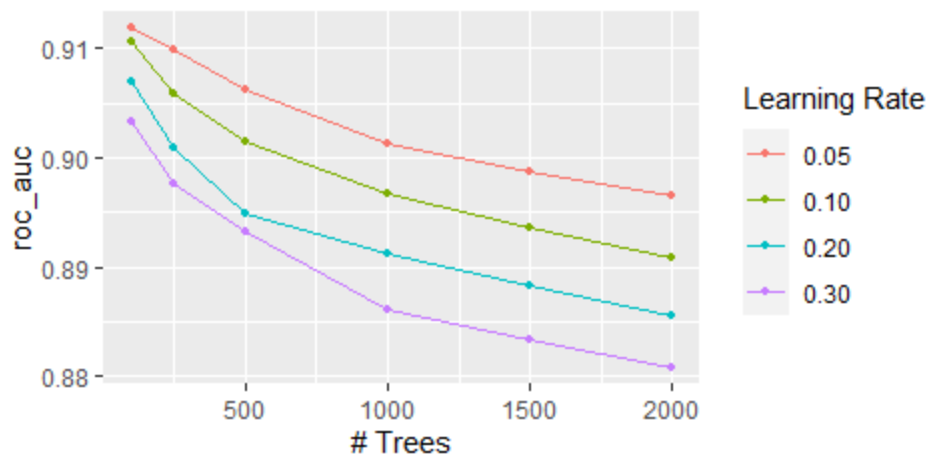
**

**Step 2- Tunning tree Deph and Minimal node size**


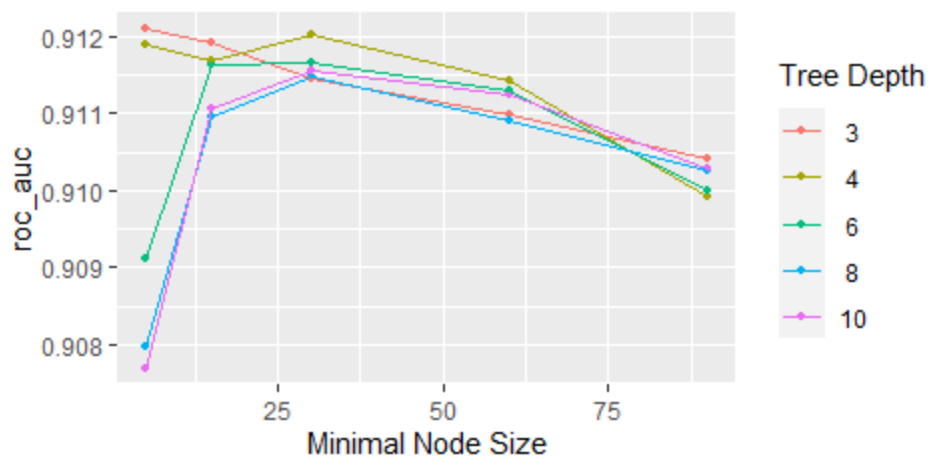


Step 3 Tunning minimal loss reduction


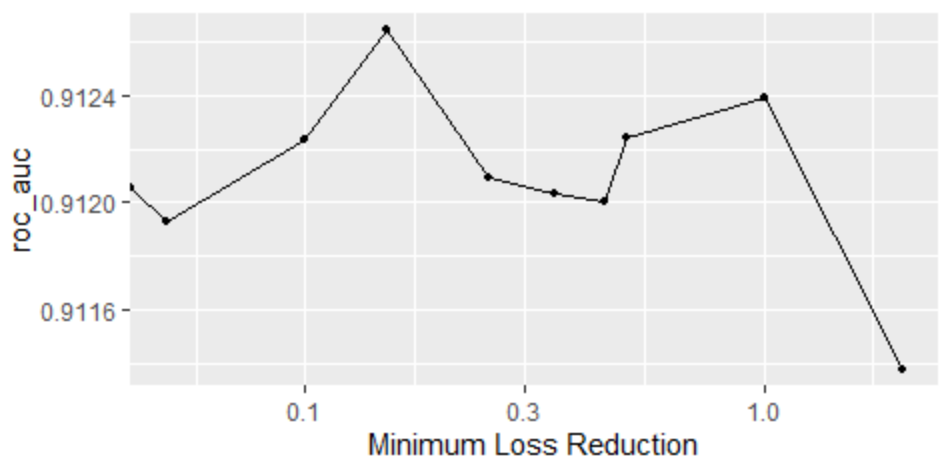


Step 4 Tunning mtry and Sample Size


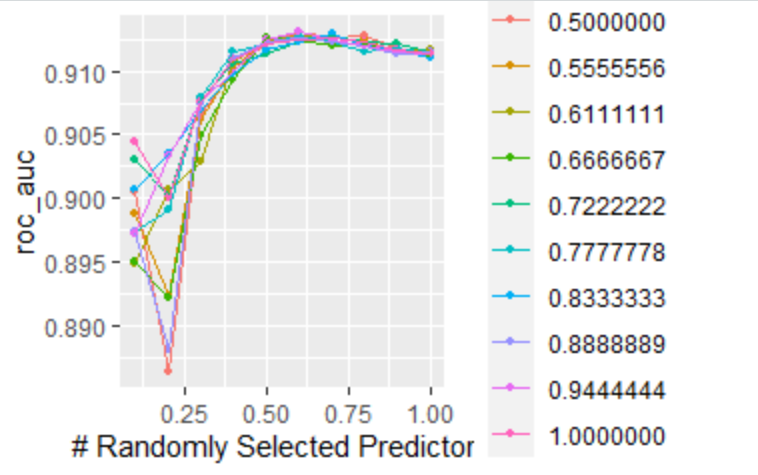


Step 5- Learning rate and trees final again


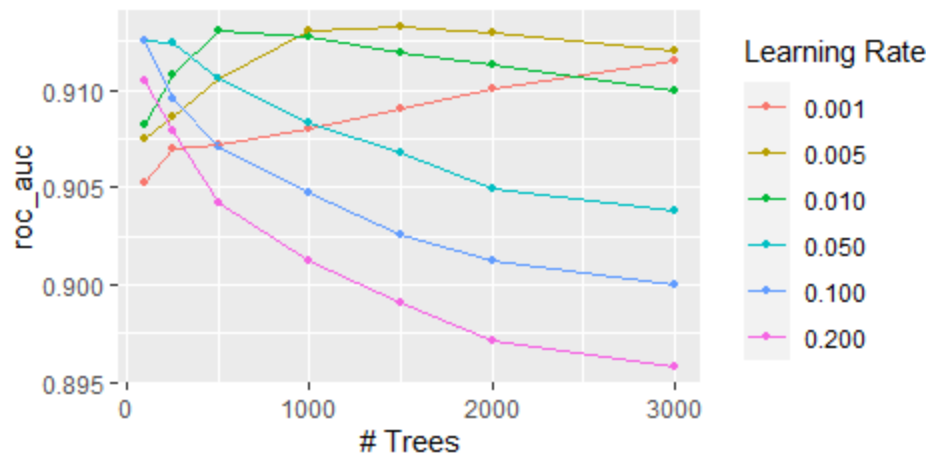


**Step 6 - Collect_metrics in the test set.**

1 sens binary 0.389 Preprocessor1_Model1

2 spec binary 0.979 Preprocessor1_Model1

3 accuracy binary 0.928 Preprocessor1_Model1

4 ppv binary 0.633 Preprocessor1_Model1

5 f_meas binary 0.482 Preprocessor1_Model1

6 npv binary 0.944 Preprocessor1_Model1

7 roc_auc binary 0.910 Preprocessor1_Model1

**Step 7 - Importance of predictors**

**
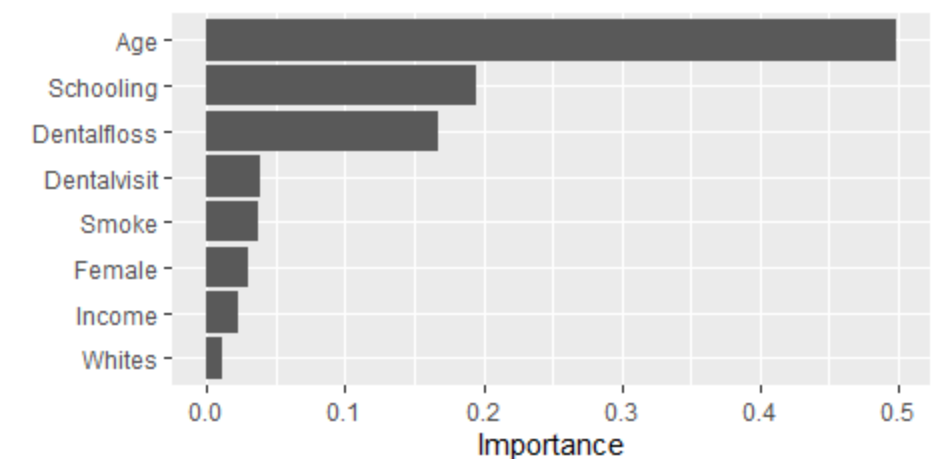
**

Step 8 – Roc –Curve


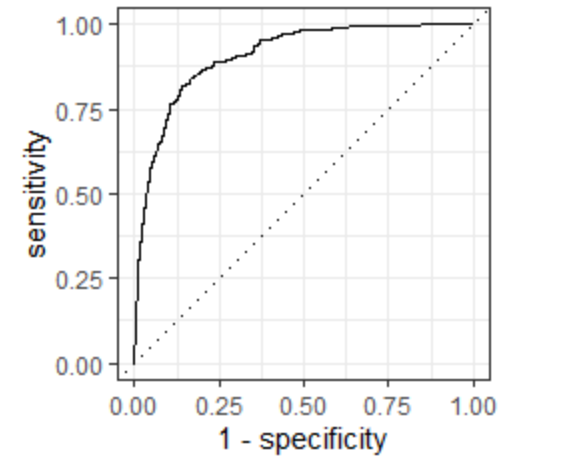


**South Region**

Step 1 - Tunning trees and learning rate


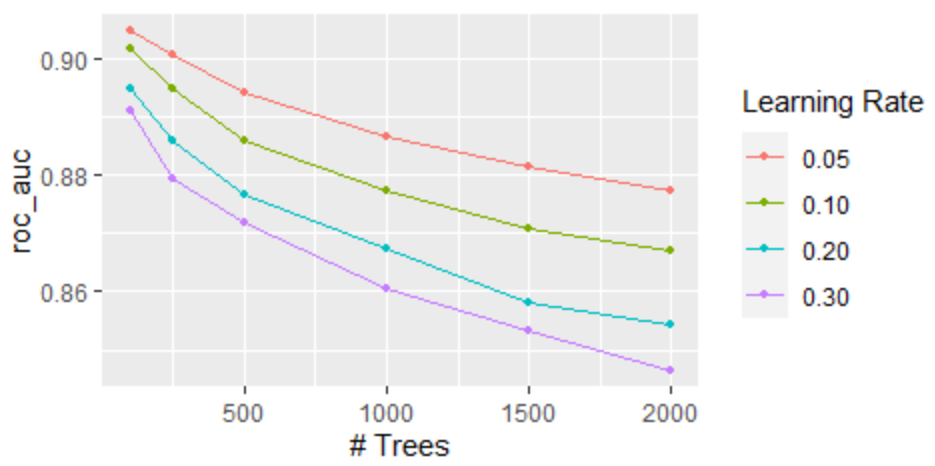


**Step 2- Tunning tree Deph and Minimal node size**

**
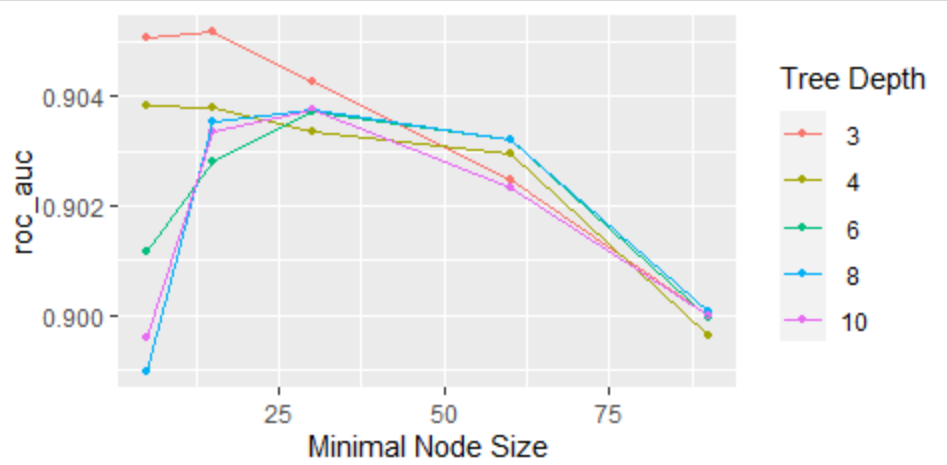
**

Step 3 Tunning minimal loss reduction


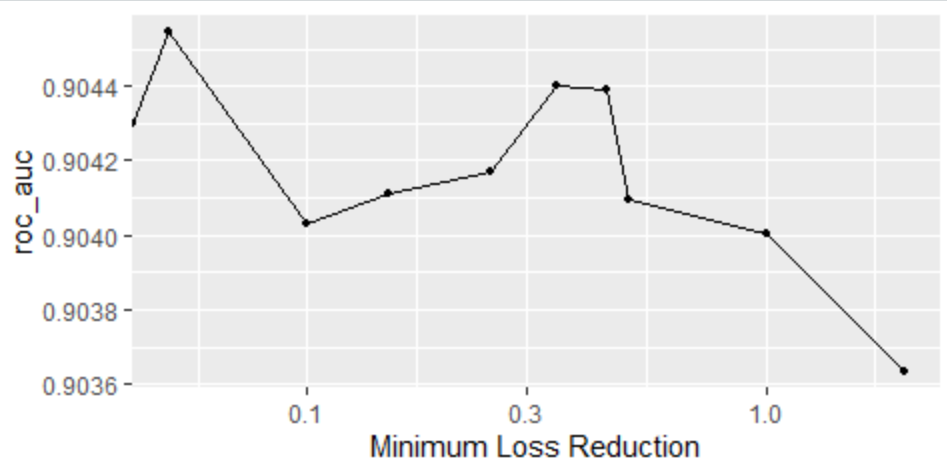


Step 4 Tunning mtry and Sample Size


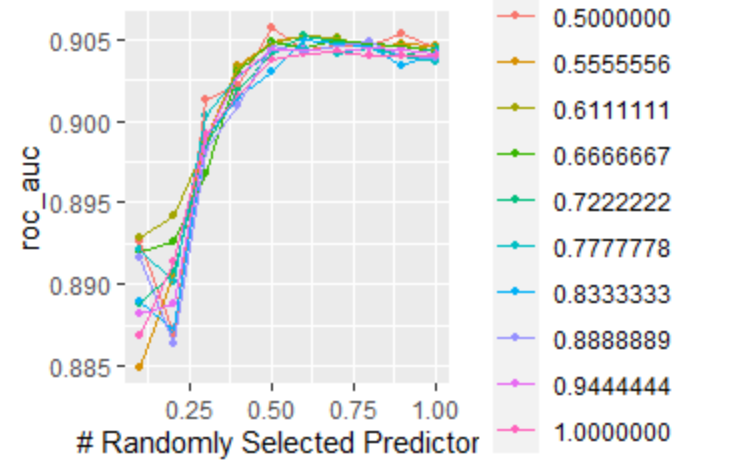


Step 5- Learning rate and trees final again


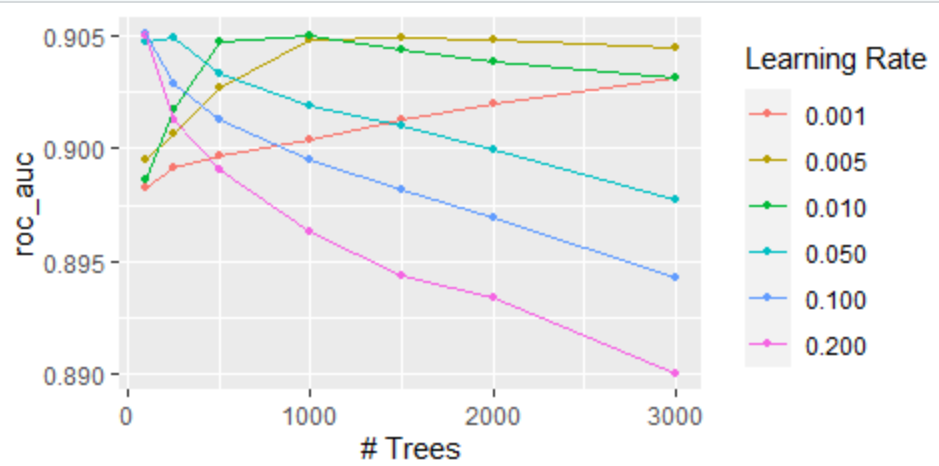


**Step 6 - Collect_metrics in the test set.**

1 sens binary 0.388 Preprocessor1_Model1

2 spec binary 0.973 Preprocessor1_Model1

3 accuracy binary 0.908 Preprocessor1_Model1

4 ppv binary 0.649 Preprocessor1_Model1

5 f_meas binary 0.486 Preprocessor1_Model1

6 npv binary 0.926 Preprocessor1_Model1

7 roc_auc binary 0.905 Preprocessor1_Model1

**Step 7 - Importance of predictors**

**
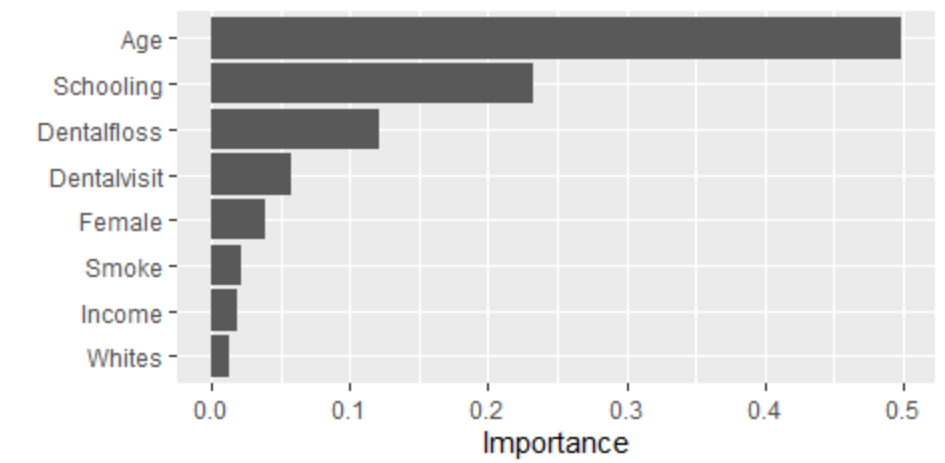
**

Step 8 – Roc –Curve


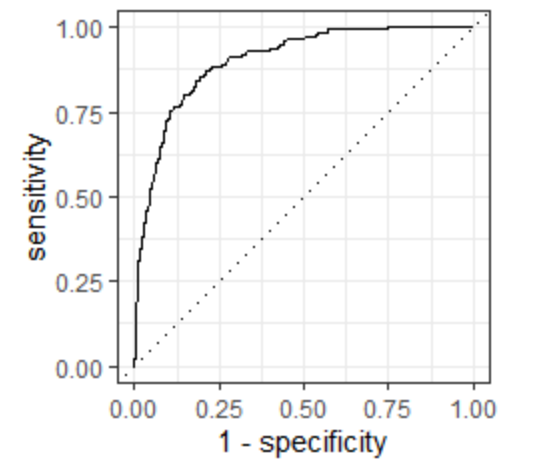


**Midwest Region**

Step 1 - Tunning trees and learning rate


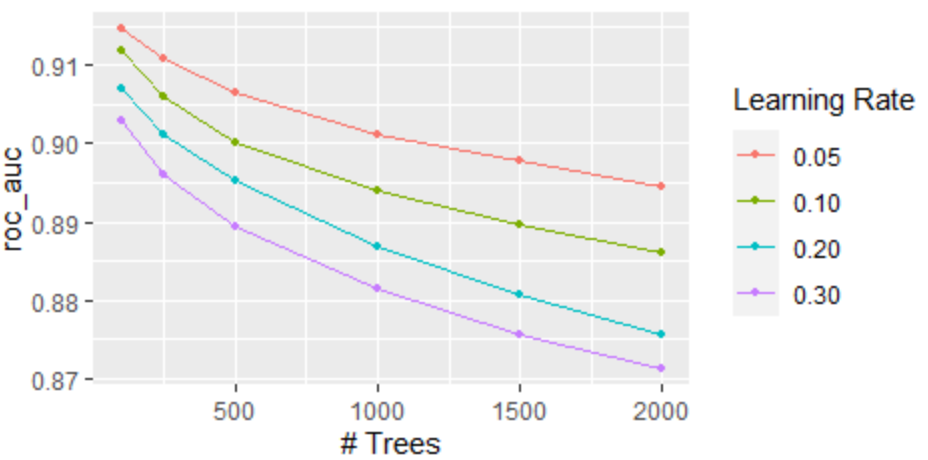


**Step 2- Tunning tree Deph and Minimal node size**

**
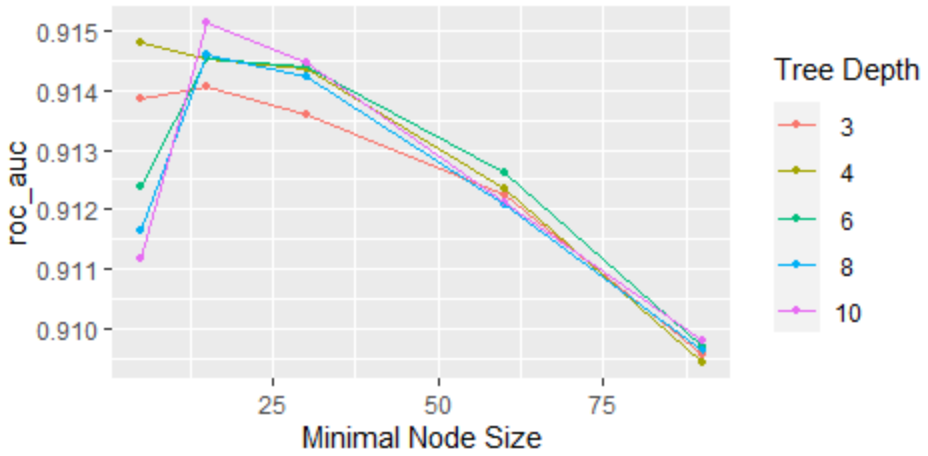
**

Step 3 Tunning minimal loss reduction


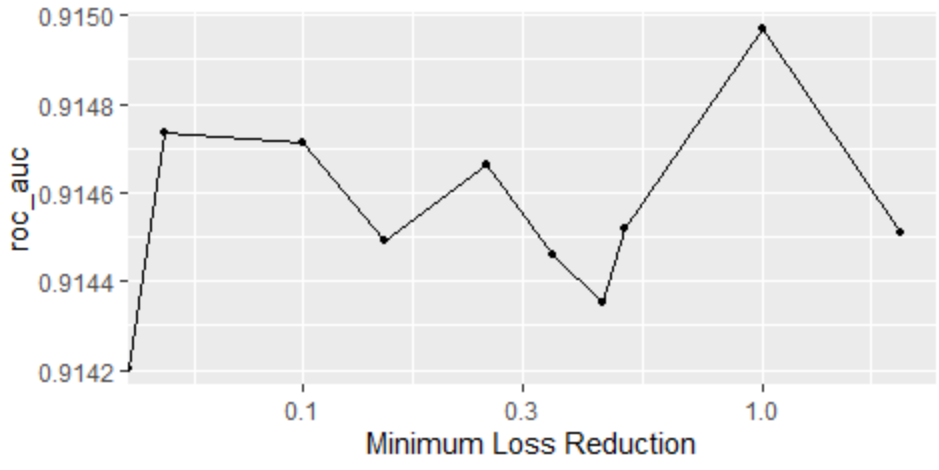


Step 4 Tunning mtry and Sample Size


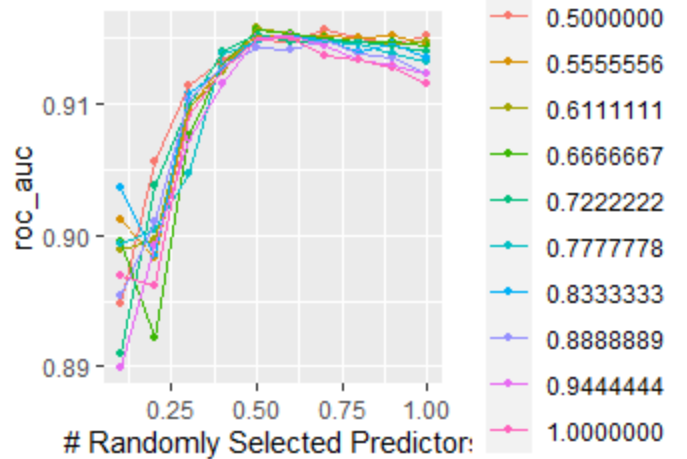


Step 5- Learning rate and trees final again


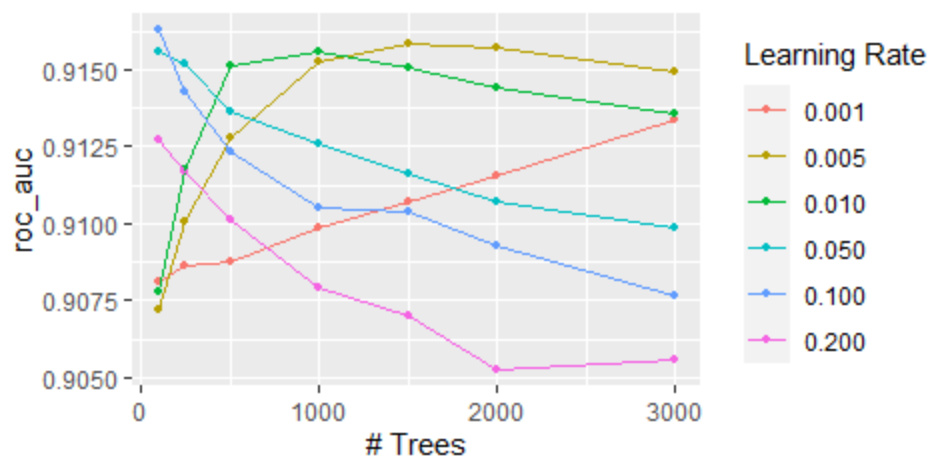


**Step 6 - Collect_metrics in the test set.**

1 sens binary 0.297 Preprocessor1_Model1

2 spec binary 0.970 Preprocessor1_Model1

3 accuracy binary 0.9 Preprocessor1_Model1

4 ppv binary 0.531 Preprocessor1_Model1

5 f_meas binary 0.381 Preprocessor1_Model1

6 npv binary 0.923 Preprocessor1_Model1

7 roc_auc binary 0.901 Preprocessor1_Model1

**Step 7 - Importance of predictors**

**
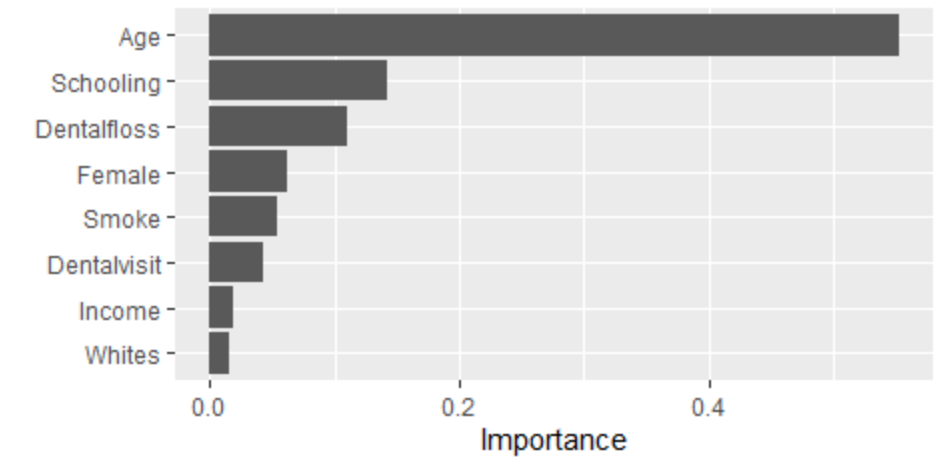
**

Step 8 – Roc –Curve


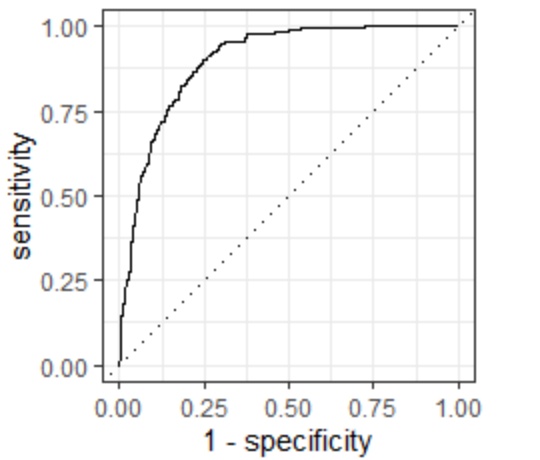


**Northeast region**

Step 1 - Tunning trees and learning rate


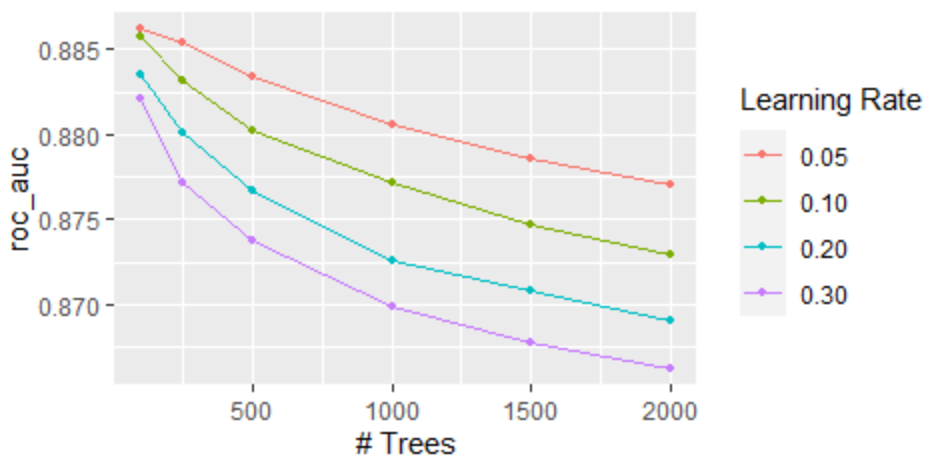


**Step 2- Tunning tree Deph and Minimal node size**

**
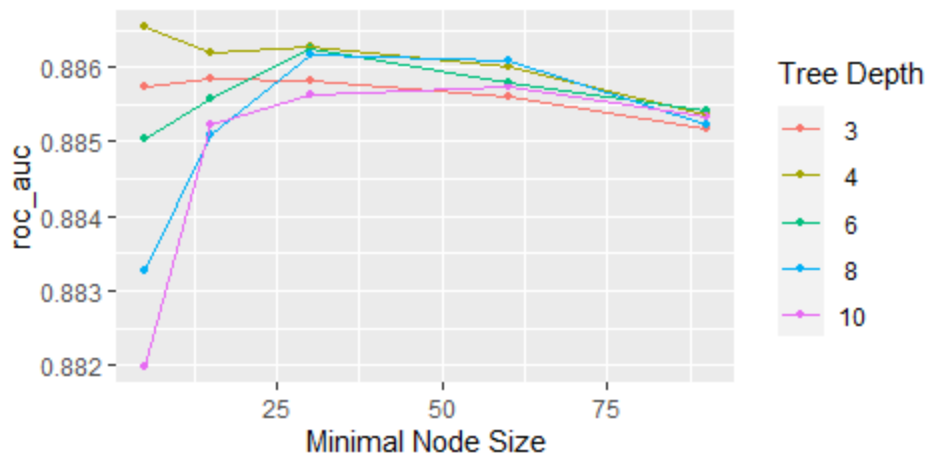
**

Step 3 Tunning minimal loss reduction


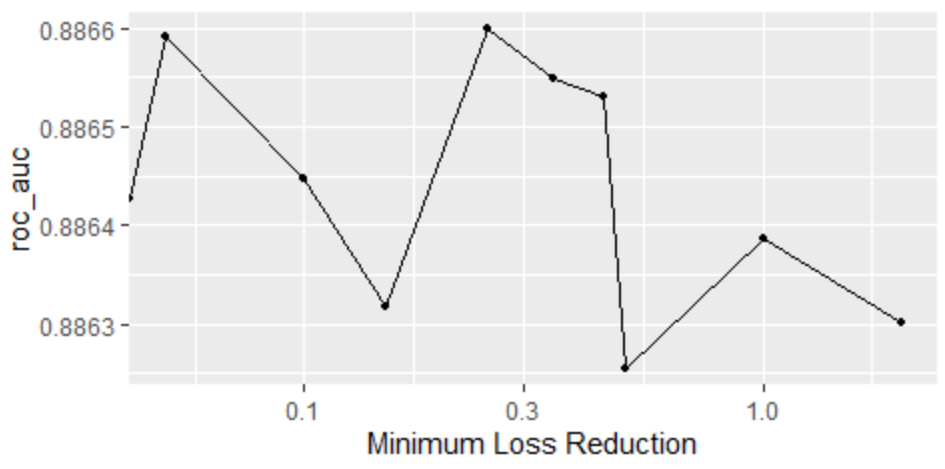


Step 4 Tunning mtry and Sample Size


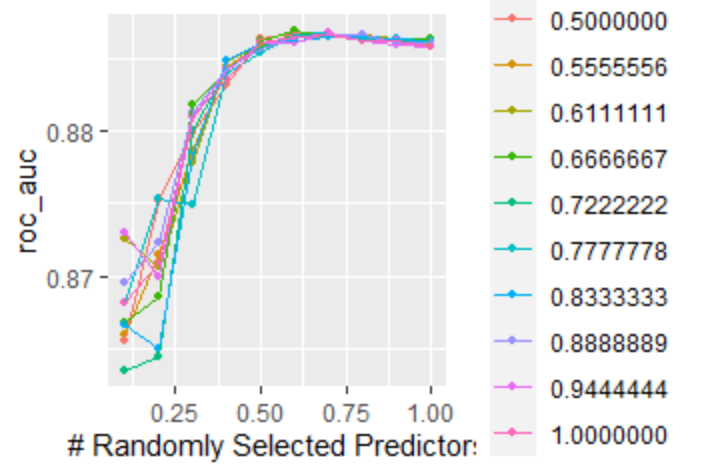


Step 5- Learning rate and trees final again


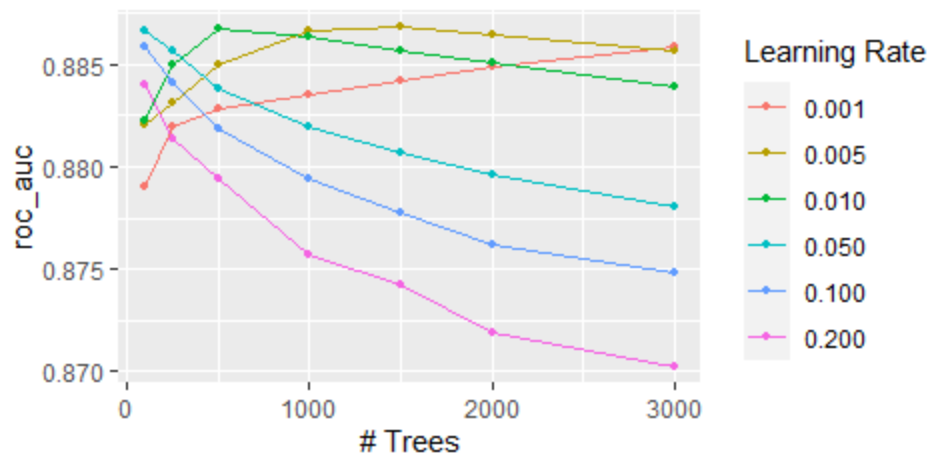


**Step 6 – Collect metrics in the test set.**

1 sens binary 0.412 Preprocessor1_Model1

2 spec binary 0.956 Preprocessor1_Model1

3 accuracy binary 0.875 Preprocessor1_Model1

4 ppv binary 0.620 Preprocessor1_Model1

5 f_meas binary 0.495 Preprocessor1_Model1

6 npv binary 0.903 Preprocessor1_Model1

7 roc_auc binary 0.884 Preprocessor1_Model1

**Step 7 - Importance of predictors**

**
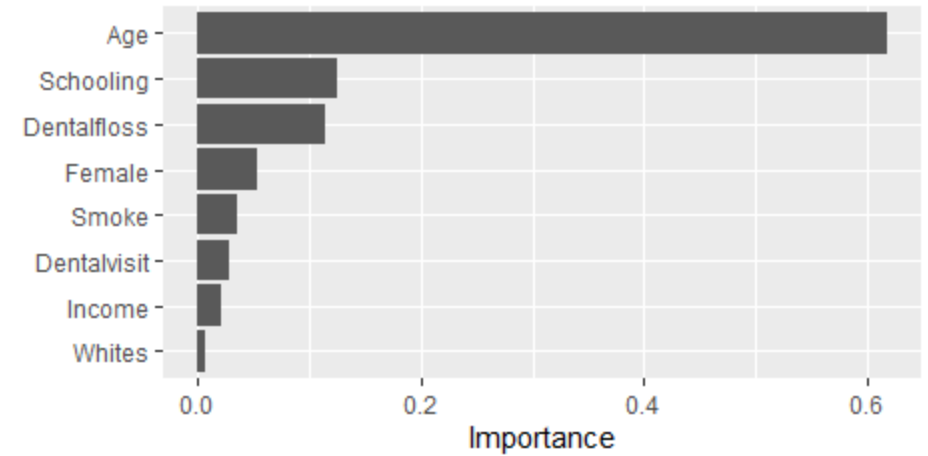
**

Step 8 – Roc –Curve


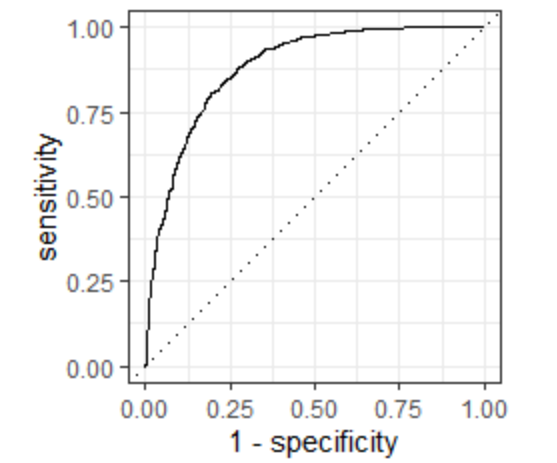


**Older Adults**

**Southeast region**

Step 1 - Tunning trees and learning rate

**
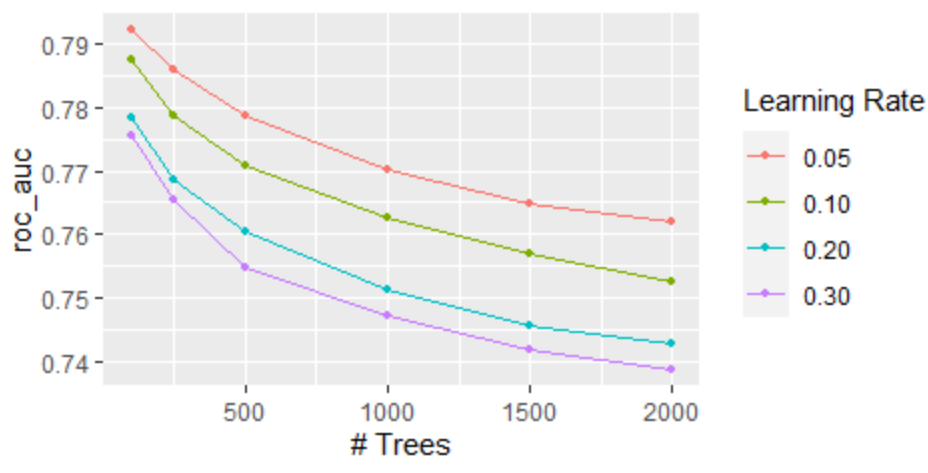
**

Step 2 Tunning tree Deph and Minimal node size


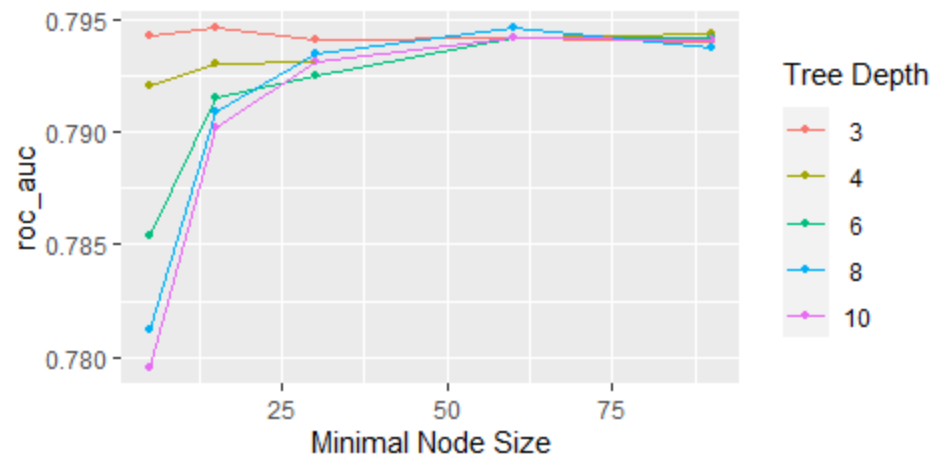


Step 3 tunning minimal loss reduction


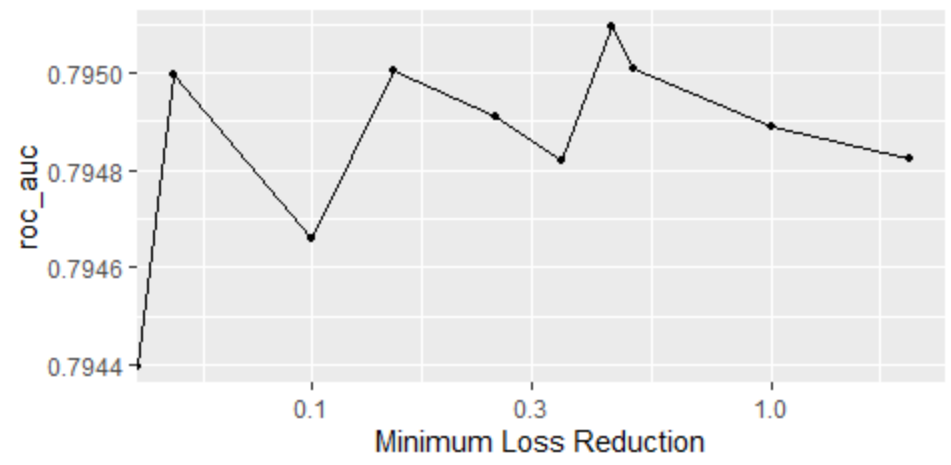


Step 4 Tunning mtry and Sample Size


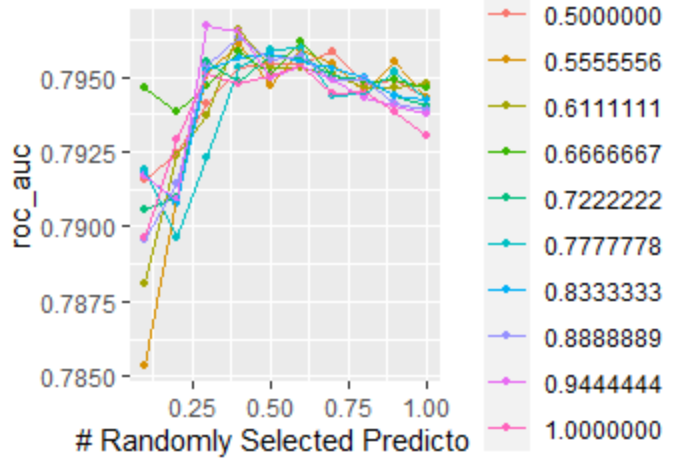


Step 5- Learning rate and trees final again


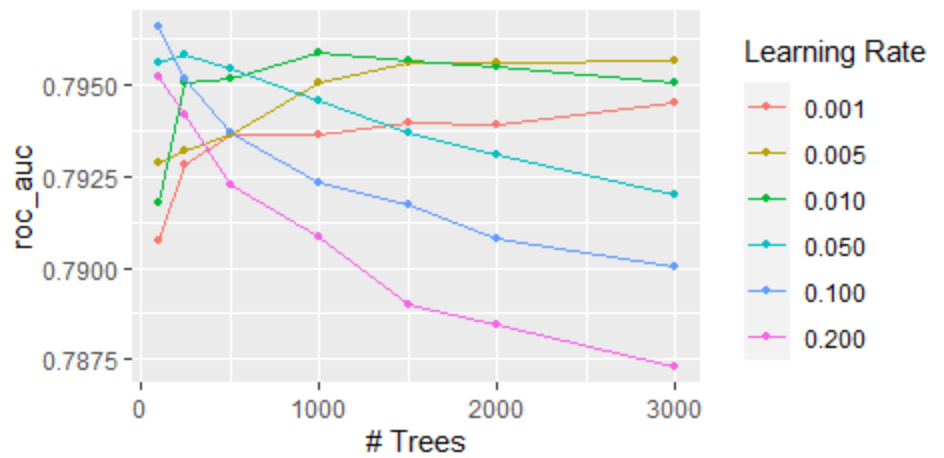


**Step 6 - Collect_metrics in the test set.**

1 accuracy binary 0.727 Preprocessor1_Model1

2 f_meas binary 0.760 Preprocessor1_Model1

3 ppv binary 0.740 Preprocessor1_Model1

4 npv binary 0.709 Preprocessor1_Model1

5 sens binary 0.782 Preprocessor1_Model1

6 spec binary 0.659 Preprocessor1_Model1

7 roc_auc binary 0.799 Preprocessor1_Model1

**Step 7 - Importance of predictors**

**
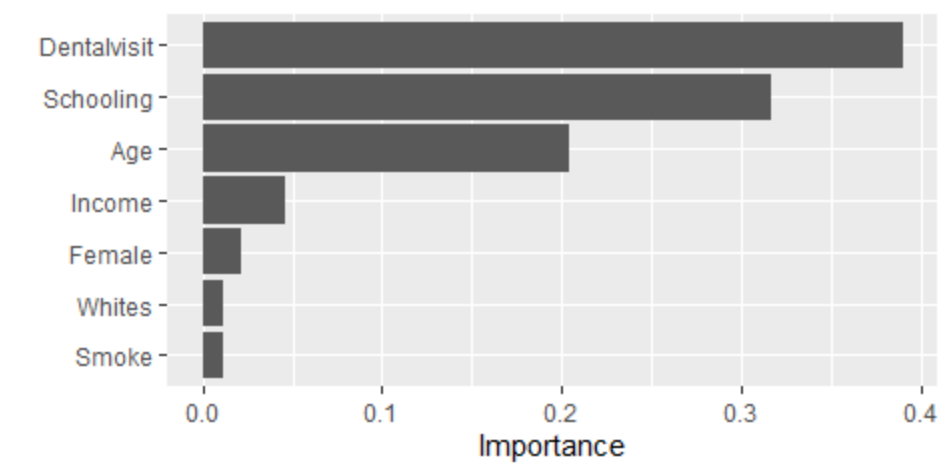
**

**Step 8 – Roc –Curve**

**
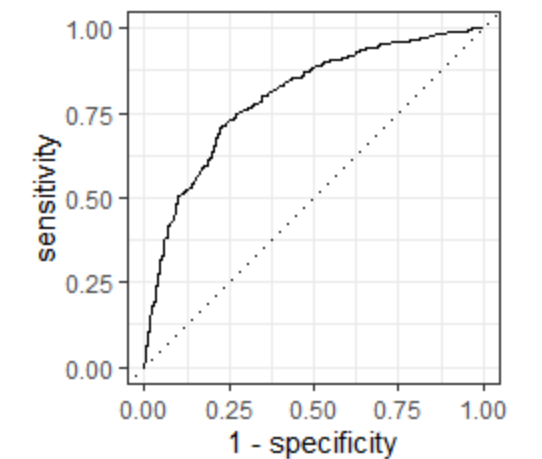
**

**South Region**

Step 1 - Tunning trees and learning rate


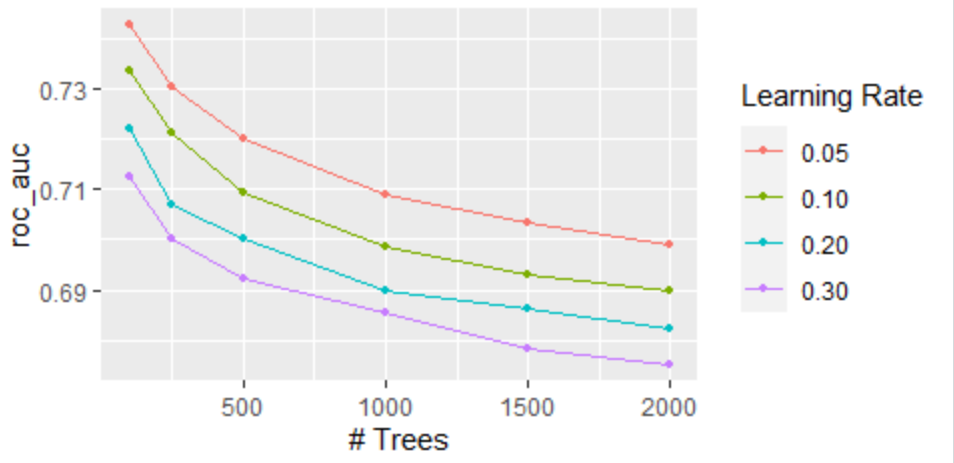


Step 2 Tunning tree Deph and Minimal node size


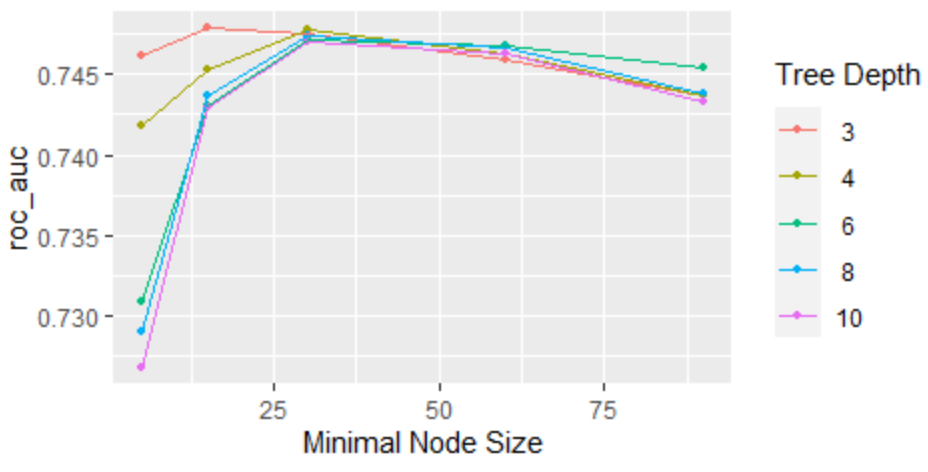


Step 3 tunning minimal loss reduction


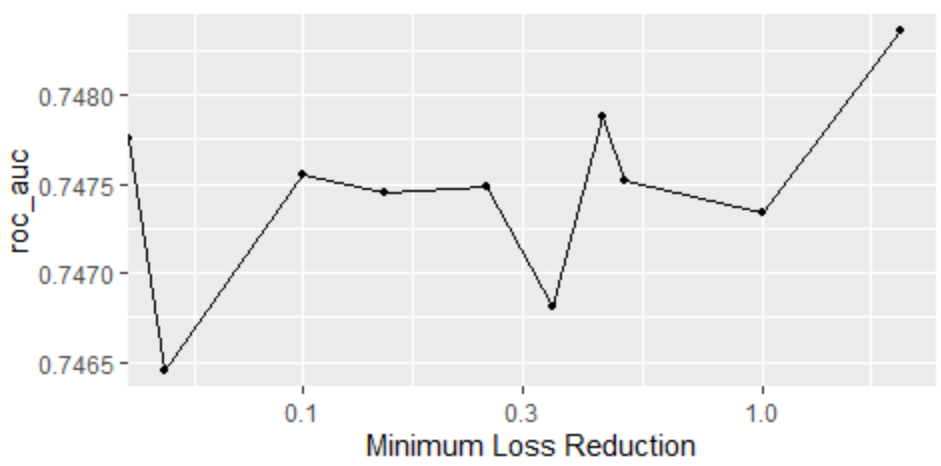


Step 4 Tunning mtry and Sample Size


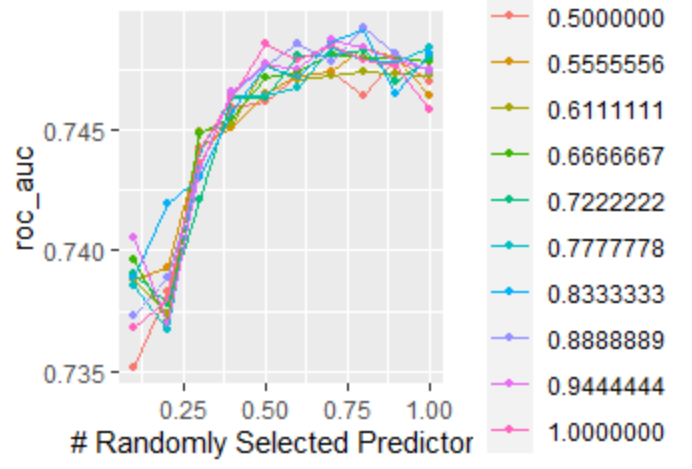


Step 5- Learning rate and trees final again


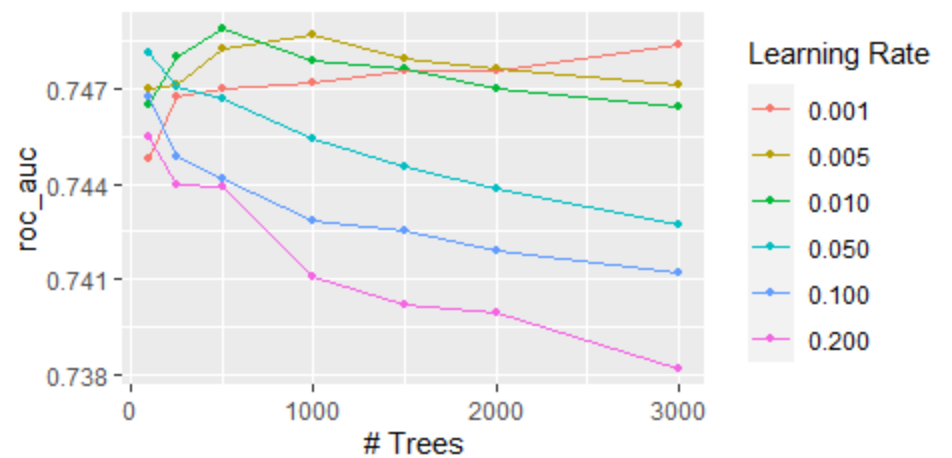


**Step 6 - Collect_metrics in the test set.**

1 accuracy binary 0.711 Preprocessor1_Model1

2 f_meas binary 0.755 Preprocessor1_Model1

3 ppv binary 0.717 Preprocessor1_Model1

4 npv binary 0.701 Preprocessor1_Model1

5 sens binary 0.799 Preprocessor1_Model1

6 spec binary 0.599 Preprocessor1_Model1

7 roc_auc binary 0.770 Preprocessor1_Model1

**Step 7 - Importance of predictors**

**
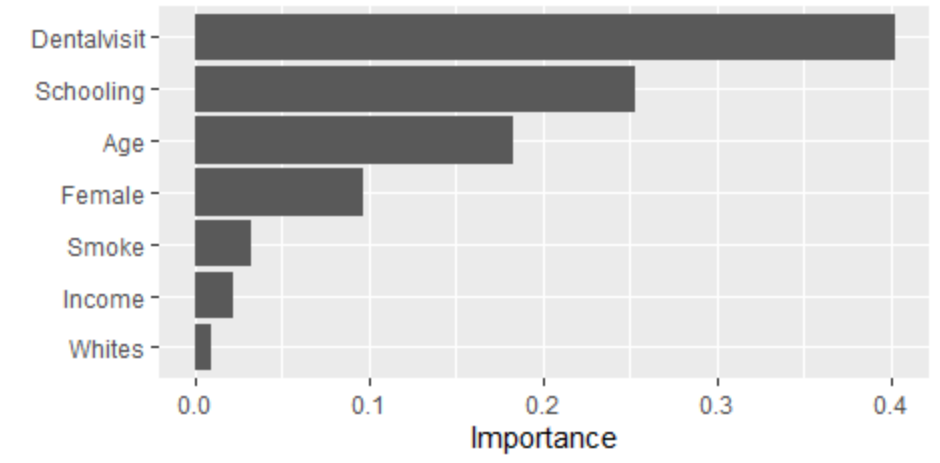
**

**Step 8 – Roc –Curve**

**
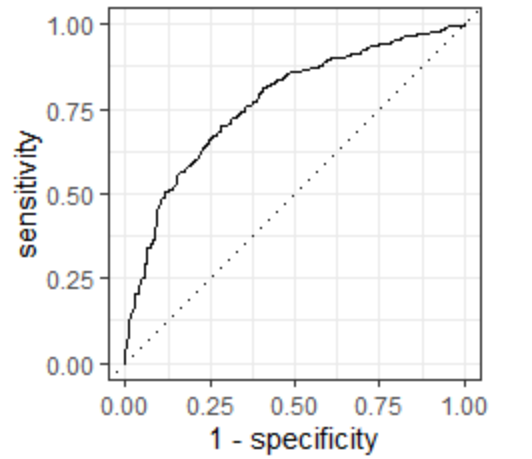
**

**Midwest Region**

Step 1 - Tunning trees and learning rate


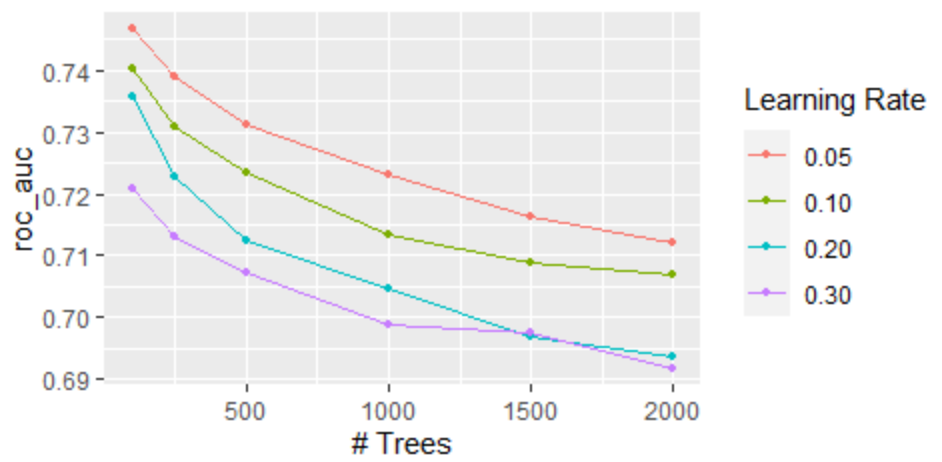


Step 2 Tunning tree Deph and Minimal node size


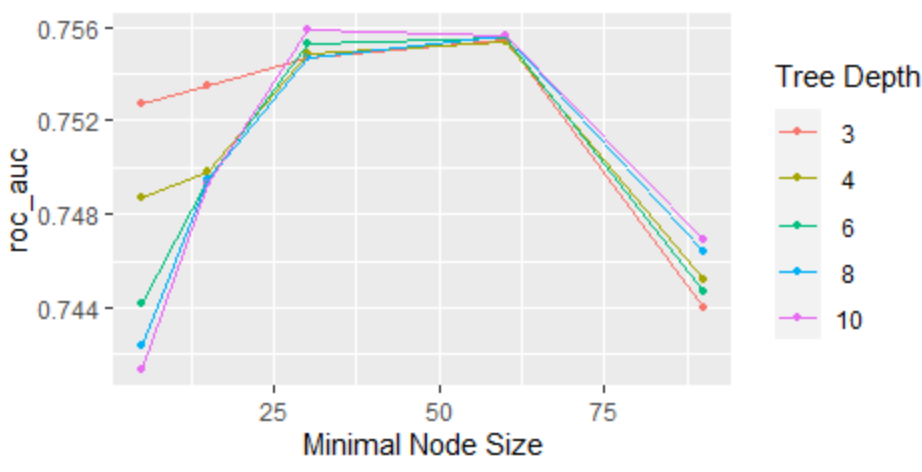


Step 3 tunning minimal loss reduction


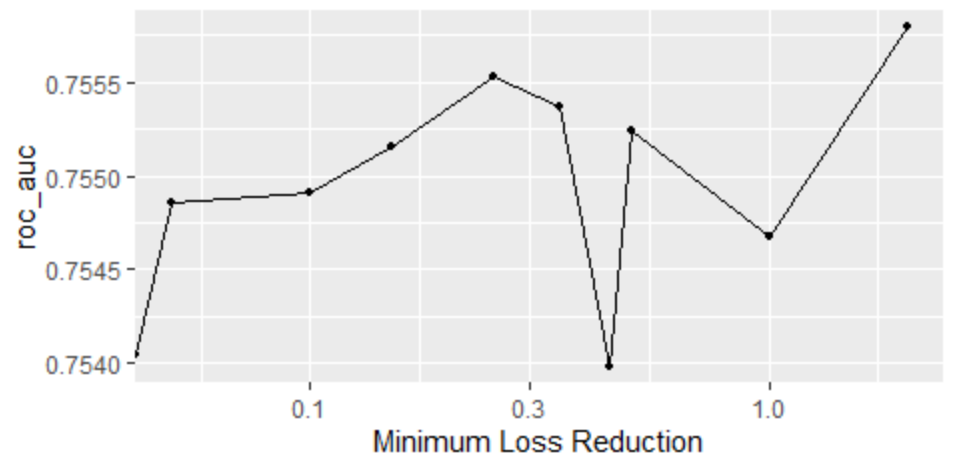


Step 4 Tunning mtry and Sample Size


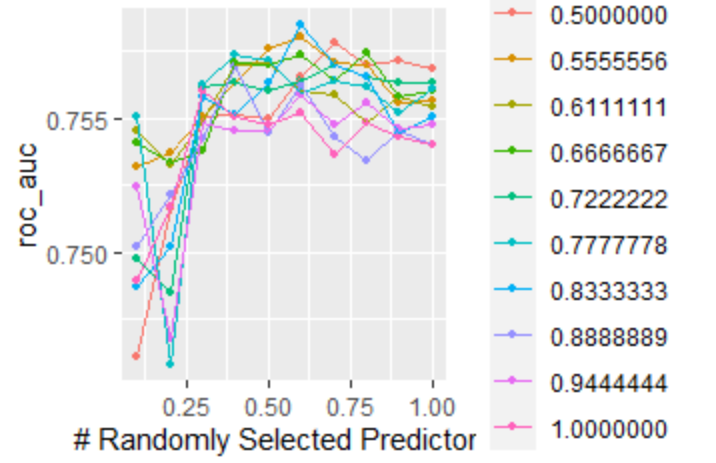


Step 5- Learning rate and trees final again


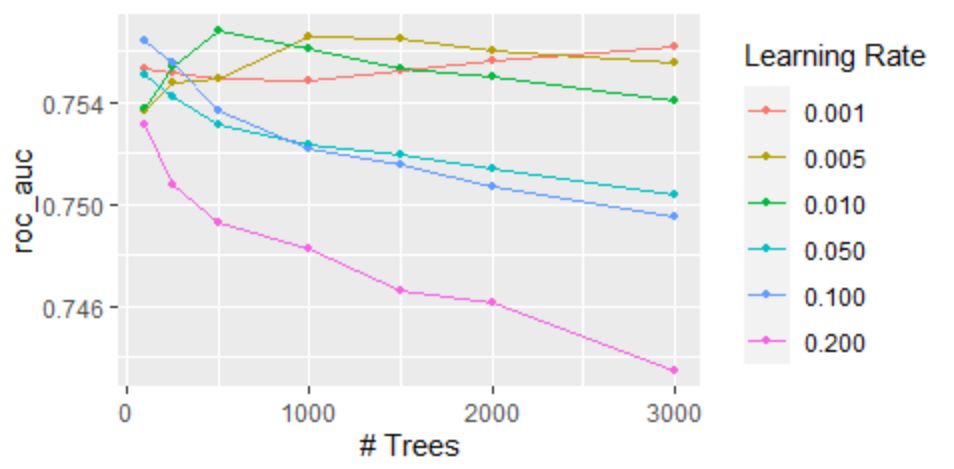


**Step 6 - Collect_metrics in the test set.**

1 accuracy binary 0.693 Preprocessor1_Model1

2 f_meas binary 0.712 Preprocessor1_Model1

3 ppv binary 0.705 Preprocessor1_Model1

4 npv binary 0.678 Preprocessor1_Model1

5 sens binary 0.719 Preprocessor1_Model1

6 spec binary 0.663 Preprocessor1_Model1

7 roc_auc binary 0.761 Preprocessor1_Model1

**Step 7 - Importance of predictors**

**
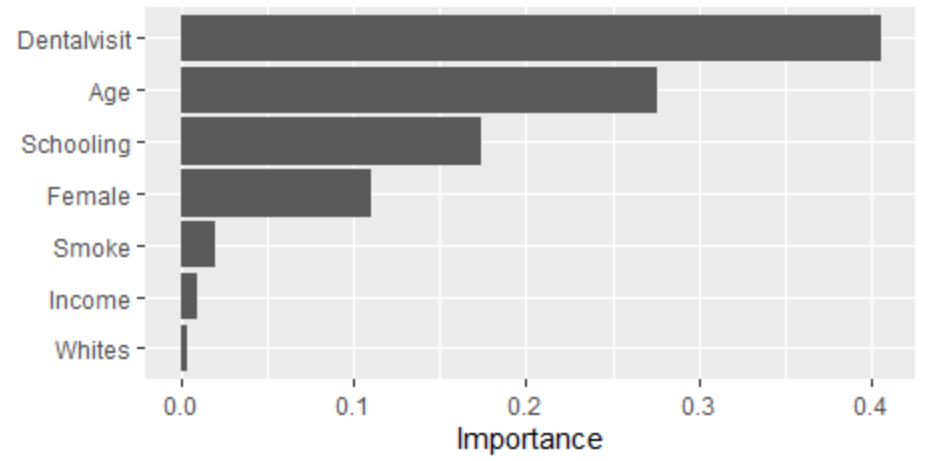
**

**Step 8 – Roc –Curve**

**
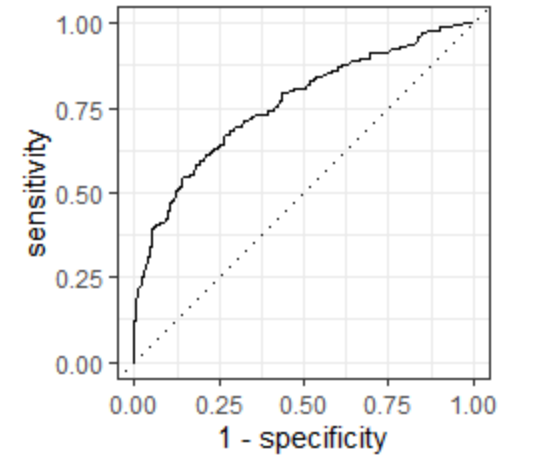
**

**Northeast Region**

**Step 1 - Tunning trees and learning rate**


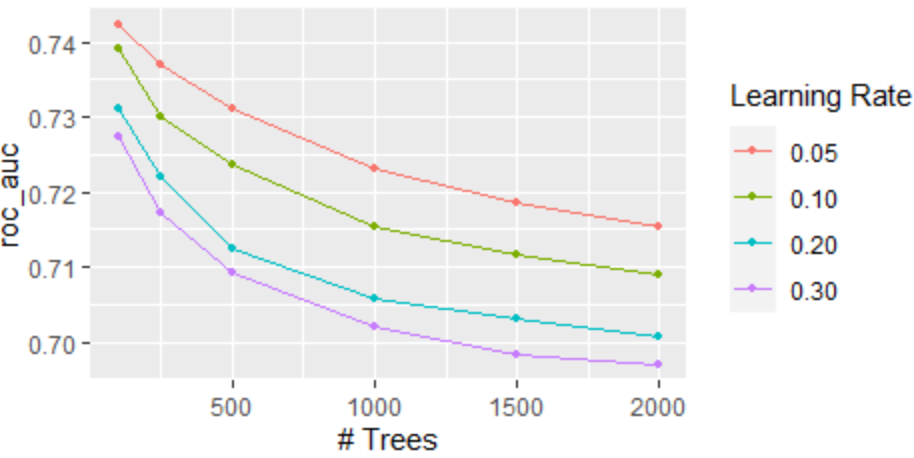


Step 2 Tunning tree Deph and Minimal node size


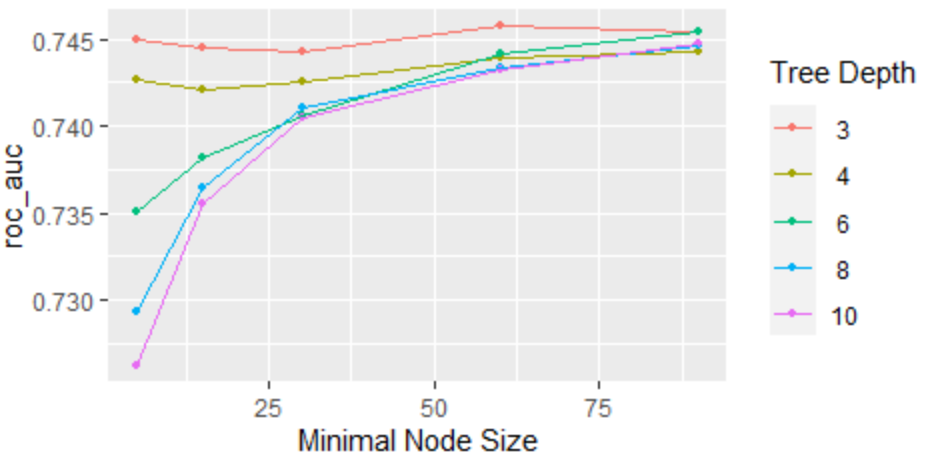


Step 3 tunning minimal loss reduction


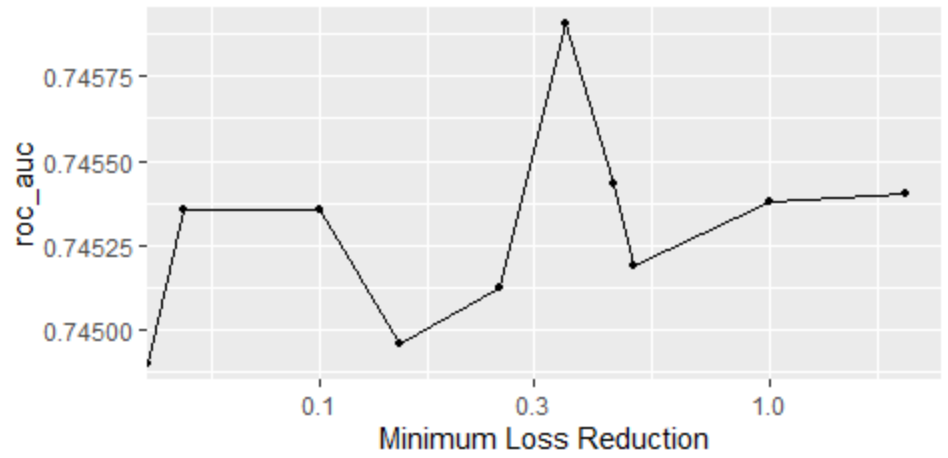


Step 4 Tunning mtry and Sample Size


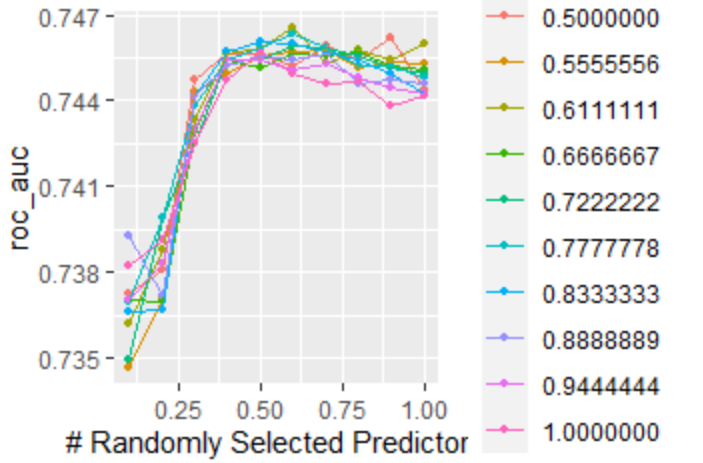


Step 5- Learning rate and trees final again


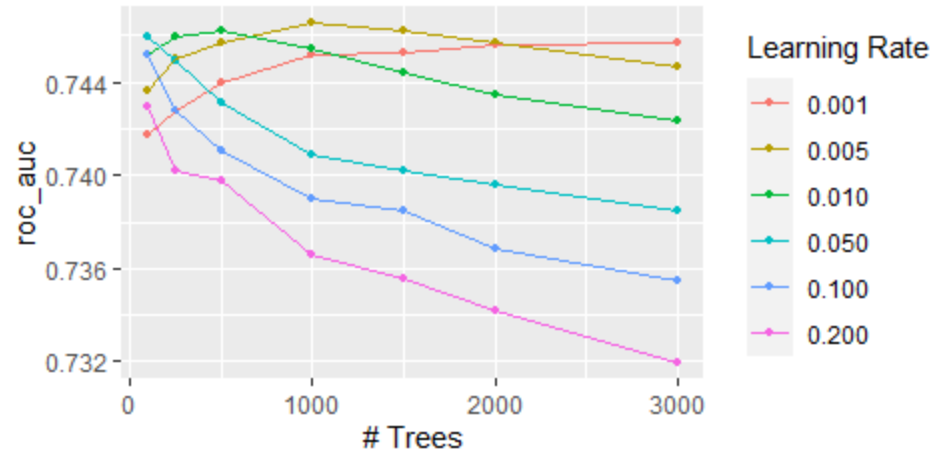


**Step 6 - Collect_metrics in the test set.**

1 accuracy binary 0.709 Preprocessor1_Model1

2 f_meas binary 0.637 Preprocessor1_Model1

3 ppv binary 0.690 Preprocessor1_Model1

4 npv binary 0.721 Preprocessor1_Model1

5 sens binary 0.592 Preprocessor1_Model1

6 spec binary 0.798 Preprocessor1_Model1

7 roc_auc binary 0.752 Preprocessor1_Model1

**Step 7 - Importance of predictors**

**
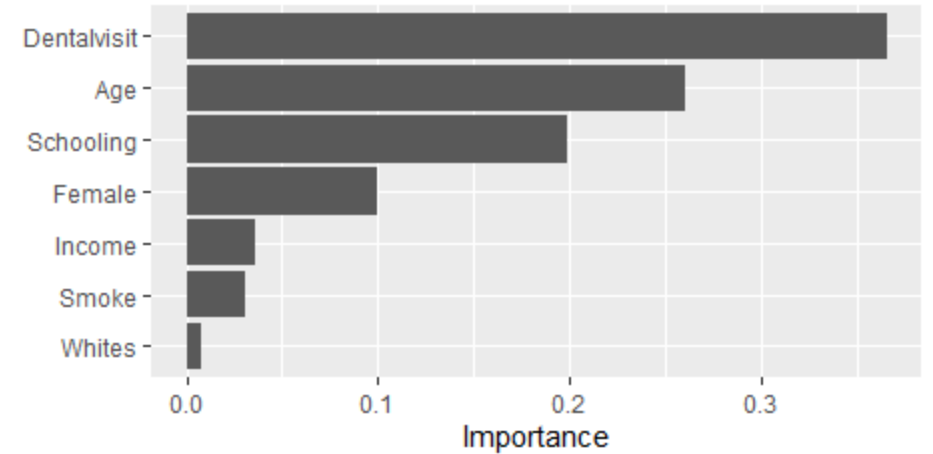
**

**Step 8 – Roc –Curve**

**
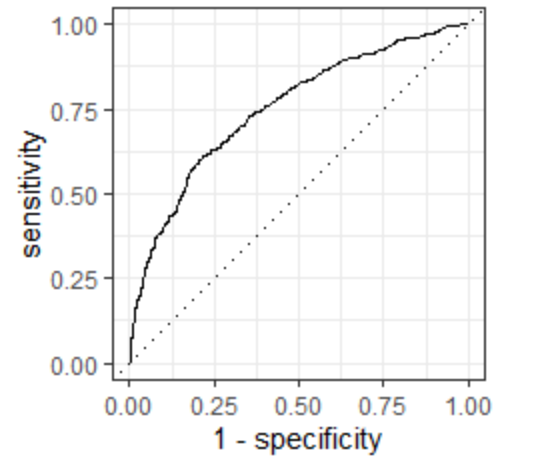
**

**North Region**

Step 1 - Tunning trees and learning rate


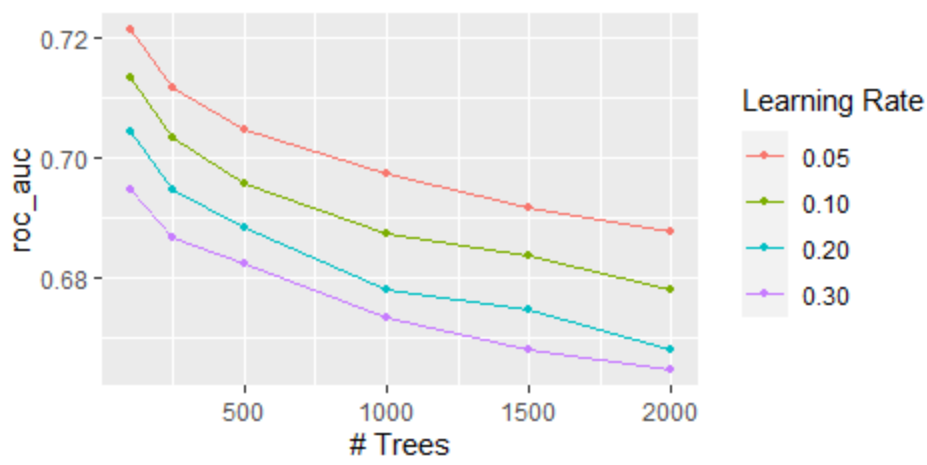


Step 2 Tunning tree Deph and Minimal node size


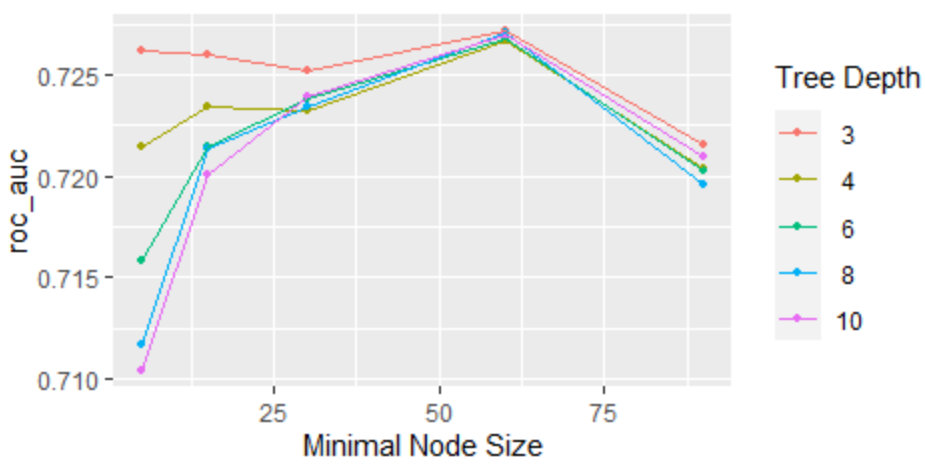


Step 3 tunning minimal loss reduction


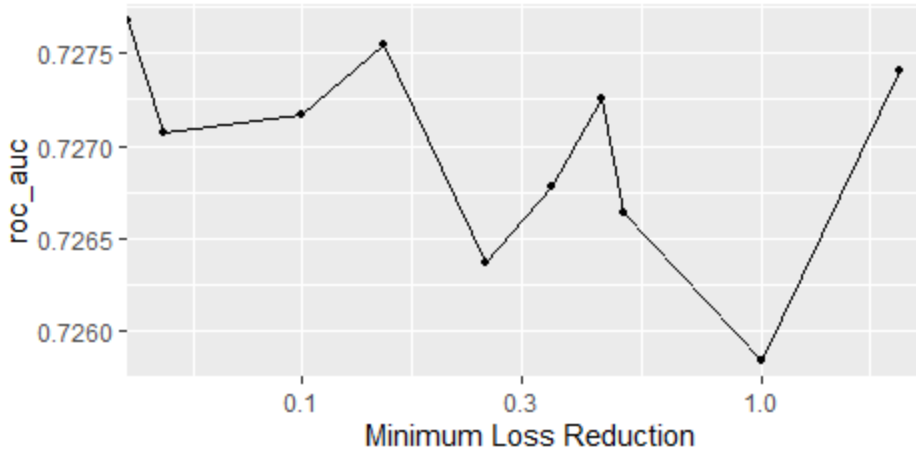


Step 4 Tunning mtry and Sample Size


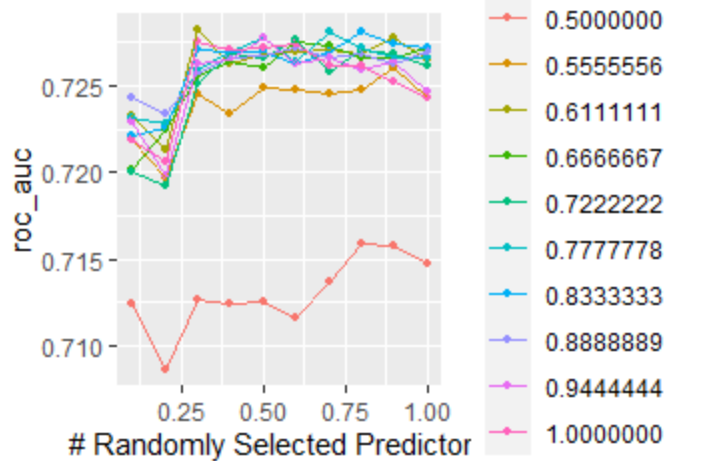


Step 5- Learning rate and trees final again


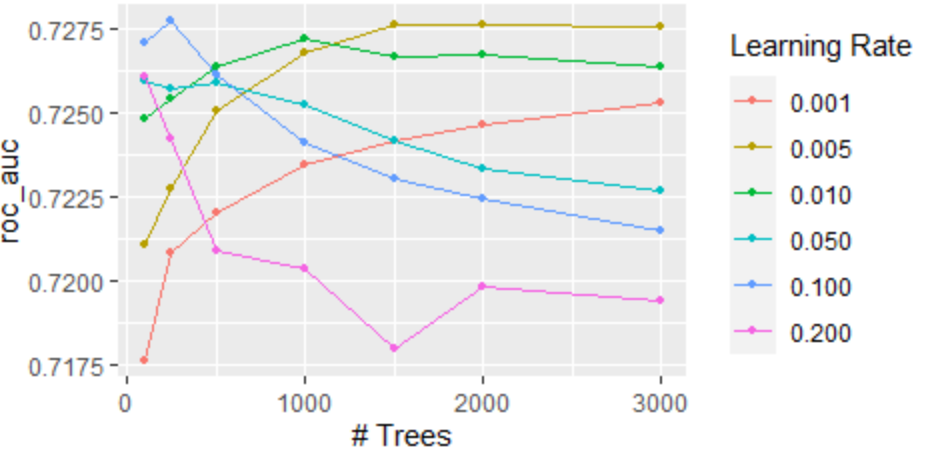


**Step 6 - Collect_metrics in the test set.**

1 accuracy binary 0.668 Preprocessor1_Model1

2 f_meas binary 0.621 Preprocessor1_Model1

3 ppv binary 0.673 Preprocessor1_Model1

4 npv binary 0.665 Preprocessor1_Model1

5 sens binary 0.577 Preprocessor1_Model1

6 spec binary 0.75 Preprocessor1_Model1

7 roc_auc binary 0.711 Preprocessor1_Model1

**Step 7 - Importance of predictors**

**
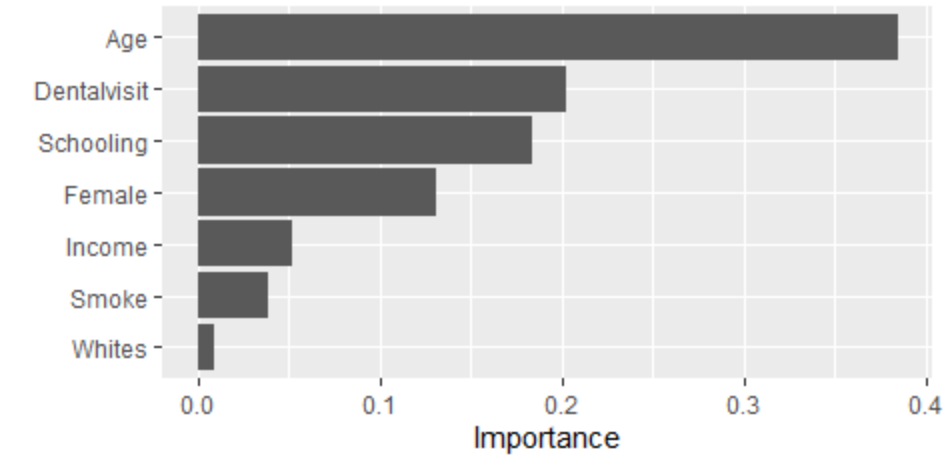
**

**Step 8 – Roc –Curve**

**
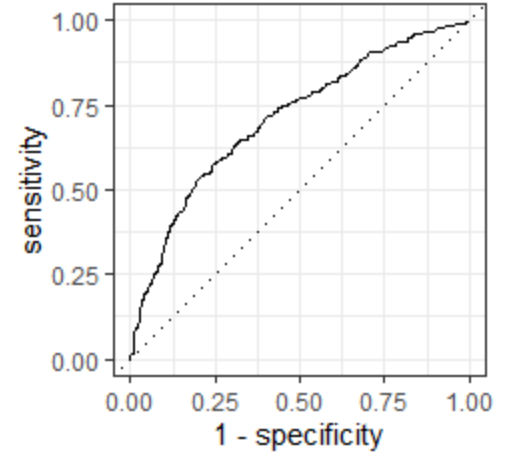
**
